# The association between early life mental health and alcohol use behaviours in adulthood: A systematic review

**DOI:** 10.1371/journal.pone.0228667

**Published:** 2020-02-18

**Authors:** Ke Ning, Dawid Gondek, Praveetha Patalay, George B. Ploubidis

**Affiliations:** Centre for Longitudinal Studies, Department of Social Science, University College of London, London, The United Kingdom; University of the Witwatersrand, SOUTH AFRICA

## Abstract

This systematic review aims to summarise current evidence on the association between early life mental health and alcohol use behaviours in adulthood. Peer-reviewed publications were located by searching EMBASE, Medline, PsycINFO, and the ISI Web of Science up to 31 October 2018. Prospective longitudinal studies reporting associations between externalising problems (EXT), internalising problems (INT), depression, anxiety before age 18, and alcohol use behaviours (alcohol consumption, heavy/problematic drinking, alcohol use disorder) after age 18 were included. After screening 17259 articles, 36 articles met the inclusion criteria. Information extracted included strength of associations, age when mental health and alcohol use behaviours were measured, sex differences in the association, and other sample characteristics. 103 tests in 23 articles were identified on the externalising domain and 135 tests in 26 articles on the internalising domain. 37 out of 103 tests reported positive associations between EXT and alcohol use behaviours. The likelihood of observing positive associations was higher for more severe alcohol use outcomes, but this trend disappeared among high-quality studies. Findings on associations between internalising domain and alcohol use varied across their subtypes. INT tended to be negatively associated with alcohol consumption but positively associated with more severe outcomes (heavy/problematic drinking, alcohol use disorder). Depression tended to be positively associated with alcohol outcomes, while no clear association between anxiety and alcohol outcomes was evident. Variation of the association across developmental timing, sex, culture, historical period was explored where appropriate. Great heterogeneity in the current literature calls for greater attention to view the relationship developmentally.

## Introduction

Alcohol use is a major public concern, being responsible for 3.8% of deaths worldwide in 2004 with the proportion increasing to 5.9% in 2012. The inestimable burden suffered by individuals, families, and society due to alcohol-related problems has also compelled scholars’ attention [1–3]. Identifying modifiable risk factors and the interactions among them is key to successful interventions, and with the availability of longitudinal studies, researchers can now investigate risk factors for alcohol use at various life stages.

Although mixed findings exist on whether mental health during childhood and adolescence is deteriorating in recent generations, most evidence suggests an increasing trend for internalising problems (INT), mainly in girls, and a stable if not increasing trend for externalising problems (EXT) [4–9]. Several potential mechanisms of action may link mental health to alcohol use behaviours. One dominant pathway is the externalising pathway [10,11]. It is hypothesised that children with EXT have an underlying tendency toward behavioural disinhibition [12], and thus are more likely to get involved in socially undesirable or restricted actions, particularly if they are exposed to high-risk environments [13,14]. An alternative mechanism, receiving more attention in recent years, involves the internalising pathway, which refers to using alcohol as a way of self-medication or tension reduction [15]. These mechanisms of action have been supported by accumulating empirical evidence, but not all studies found the same links; results regarding the internalising pathway are particularly ambiguous [16,17]. Some studies reported positive associations in which greater INT, depression, and anxiety are related to greater subsequent alcohol use [18,19], while others found opposite associations [20–23], or no links at all [24].

Many factors may contribute to this inconsistency [15,16]. First, the high and stable comorbidity between EXT and INT in youth [25,26] may confound the association of each with alcohol use when the other one is not well adjusted for in the model [27]. Second, differing forms of internalising problems (global indices, depression, anxiety) may represent a different pathway to alcohol use [16]. Third, inconsistency may also result from varying subtypes of alcohol use behaviours (drinking frequency and quantity, binge or heavy or problematic drinking, alcohol use disorder [AUD]) across studies [28]. Furthermore, from a developmental point of view, developmental timing, sex, culture and history are the key considerations when understanding the causes and course of alcohol use behaviours [29,30]. It matters when experiences occur and manifest in regard to their meaning, and this is true for both mental health and alcohol use behaviours. The stage when mental health problems develop or the duration of the problems may present different associations with later alcohol use [31]. Also, drinking behaviours during adolescence are largely influenced by social context (mainly peers and parents) [32–37], while drinking patterns in adulthood may be more established [38]. Robust sex differences in the prevalence of both mental health problems and alcohol use behaviours were found [39–42], however, how sex may play a role in the association between mental health and alcohol use is yet unclear, and how sex is treated in the analysis (whether male and female subjects are analysed separately, or sex is adjusted for or added as an interaction term with mental health problems) varies across studies. Historical period and context may contribute to different levels of EXT/INT and alcohol use, and more interestingly to the relationships between them [22]. Different from other drugs, alcohol is part of every culture and regulated by social norms about proper context of use and availability in society [43]. Thus, comparing associations without taking these factors into account may also contribute to the inconsistent findings.

Due to the above challenges, few systematic reviews have summarised the evidence on the association between mental health and alcohol use until recent years. One systematic review conducted by Hussong et al. (2017) summarised the association between negative affect symptoms (internalising problems, depression and anxiety) and adolescent substance use, controlling for externalising problems [16]. No consistent results were found regarding alcohol use, but the review indicated varying associations between internalising domain and substance use across their subtypes. Another systematic review studying conduct problem trajectories and a series of psychosocial outcomes examined five articles on alcohol use and found that, compared to low conduct problems, both early-onset and persistent (EOP) and adolescent-onset (AO) conduct problems were positively associated with later alcohol use (OR (95%CI): 1.85 (1.04, 3.28) for EOP and 1.72 (1.23, 2.41) for AO), but this was not the case for conduct problems limited to childhood [31]. A more recent review indicated that the association between childhood and adolescent anxiety and later alcohol use might vary with different alcohol subtypes but was generally inconsistent [28]. This study also found that the type and developmental period of the anxiety, the length of follow-up, the sample size, and the confounders the researchers adjusted for did not seem to explain the discrepant findings [28]. The above reviews focused on limited aspects of the association between early life mental health and alcohol use. For example, Hussong et al.’s paper did not differentiate among different subtypes of alcohol use and stages of INT [16]. Dyer et al.’s study differentiated among different subtypes of alcohol use behaviours, developmental periods of anxiety, and other potential factors, but not developmental periods of alcohol use and EXT [28].

Thus, this systematic review aims to summarise current evidence on the association between early life mental health and alcohol use behaviours in adulthood while taking into consideration potential factors that may affect the association. We confined alcohol use behaviours to those behaviours measured in adulthood, at or after age 18, for three reasons: alcohol use behaviours in adolescence was summarised by Hussong et al.’s review; how early life mental health problems are associated with alcohol use in adulthood, when drinking patterns have been established, may be different from that in adolescence, but no review has summarised the association pattern systematically; 18 years old is the minimum legal drinking age in most countries (116 out of 190 countries) [44]. Correspondingly, we looked only at studies in which mental health was measured before age 18 to ensure chronology and avoid reverse causality with alcohol use. We examined the association between early life mental health problems and alcohol use behaviours in adulthood by considering a) subtypes of early life mental health problems (EXT, INT, depression, anxiety), b) subtypes of alcohol use behaviours (alcohol consumption [frequency/volume], heavy/problematic drinking and AUD), c) whether EXT/INT was adjusted for accordingly, d) the developmental timing in which mental health problems occurred (childhood [before age 11 years], early-adolescence [11 to 15 years], adolescence [16 to 17.9 years]) [30], and alcohol use behaviours occurred (transition to adulthood [18 to 25 years], early-adulthood [26 to 40 years]), midlife and beyond [41 years old onwards]), e) whether the association varies across sex, history, and culture.

## Methods

### Search strategy and selection criteria

Initial searches were conducted on 4 April 2017 with an update search conducted on 31 October 2018. Four databases (EMBASE, Medline, PsycINFO, and the ISI Web of Science) were searched for publications (See S1 Table for the search strategy used for the ISI Web of Science). Results were merged and imported into Eppi-reviewer 4 for the first-round search and then EndNote X9 for the second round.

### Inclusion and exclusion criteria

#### Population and study type

Studies were restricted to prospective longitudinal designs that recruited samples from general community populations and collected information prospectively instead of retrospectively. Clinical and high-risk samples recruiting people diagnosed with specific mental/physical diseases and children of alcoholics were excluded. Experimental, clinical, cross-sectional, case-control, and time-series or econometric studies were excluded.

#### Exposure

Mental health problems were categorised into externalising problems (EXT), internalising problems (INT), depression, and anxiety. Under the externalising domain, we focused on general measures of EXT and conduct problems and did not include attention deficit hyperactivity disorder which is under the externalising domain but does not contain features that contribute to the externalising pathway [13]. We excluded studies measuring specific symptoms or traits, such as stealing or fighting. Studies with a wide age range population over age 18 were included only if the upper age boundary (two standard deviations above the mean age) was below age 18 to ensure mental health problems were measured below age 18 for the majority of the population. Studies that derived trajectories for mental health problems beyond age 18 were included only if the derived trajectories mainly reflected mental health status across childhood or adolescent (i.e., more than half of the measurement occasions occurred before age 18).

#### Outcome

We included all alcohol-specific outcomes and excluded substance use outcomes that did not explicitly represent alcohol use. For clarity, we further categorized alcohol use behaviours into three broad categories: alcohol consumption including drinking frequency/volumes; heavy/problematic drinking, including binge drinking, heavy drinking, and problematic drinking identified through well-known scales (e.g., Cut-down Annoyed Guilty Eye-open (CAGE) / Alcohol Use Disorder Identification Test (AUDIT)); AUD diagnosed based on the Diagnostic and Statistical Manual of Mental Disorders (DSM). As mentioned above, the included studies all measured alcohol use behaviour at or after age 18 as it is the minimum legal drinking age in most countries [44]; studies with a wide age range population under age 18 were included only if the lower age boundary (two standard deviations below the mean age) was at or above age 18. Studies that derived trajectories for alcohol use below age 18 were included only if the derived trajectories mainly reflected alcohol use in adulthood (i.e., more than half of the measurement occasions occurred after age 18). In addition, all studies included in this review had alcohol outcomes that were measured at least one year after the mental health measurements were taken to reflect the long-term prospective association between them.

### Screening and data extraction

Guidelines set forth by the Preferred Reporting Items for Systematic Reviews and Meta-Analyses (PRISMA) were followed to ensure transparency [45], and the protocol for this systematic review was published on PROSPERO (registration number: CRD42018115502).

**Fig 1:**
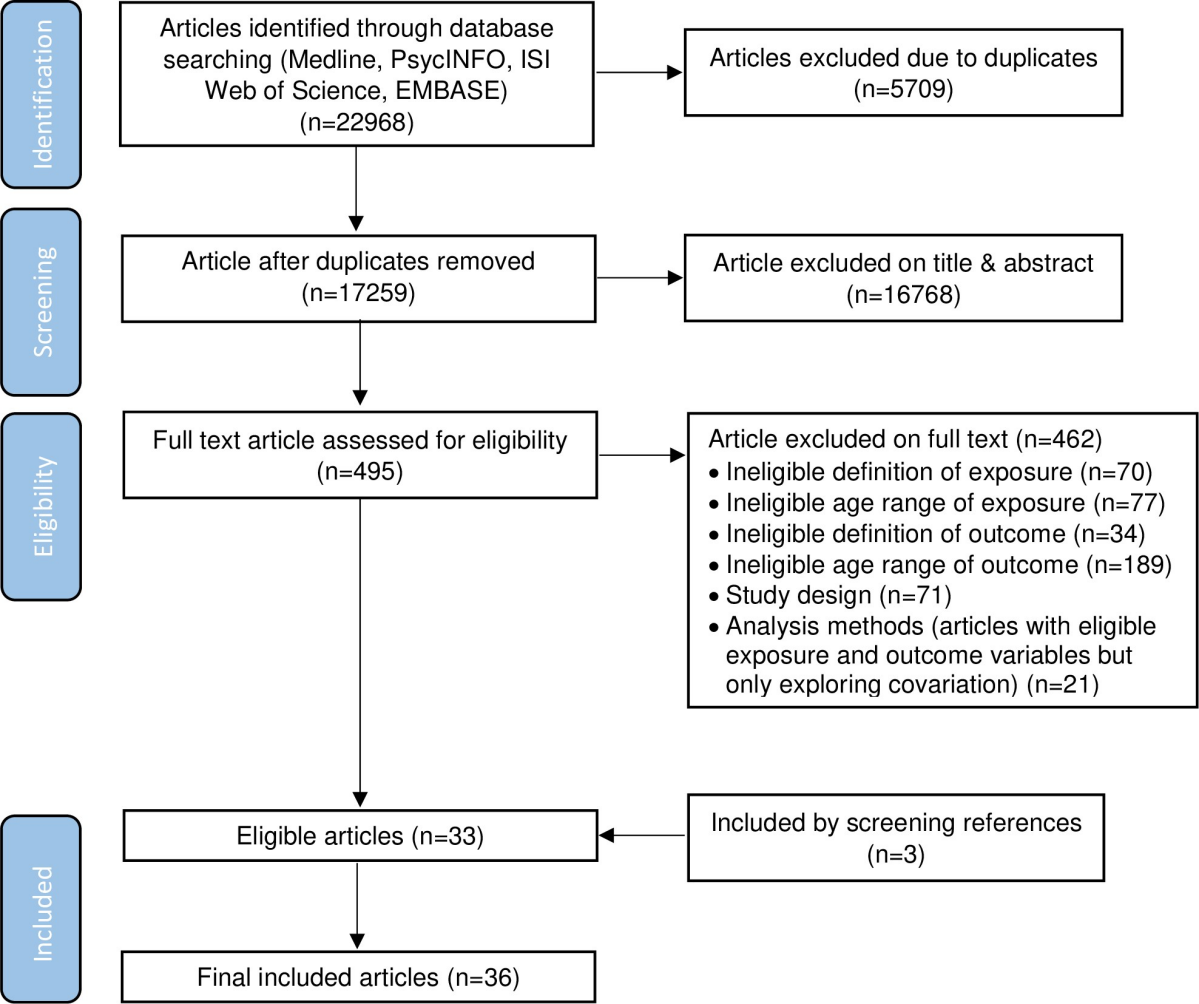
Flow chart of the screening process.

After excluding 5833 duplicates, 17,259 articles were screened for inclusion, and 15% of them were independently screened by DG. The agreement rate was 97.9% and Cohen’s kappa was 0.71 at this stage. Disagreements were discussed and consensus was reached before screening the rest of the articles. After excluding 16768 articles based on the titles and abstracts, 495 articles were retrieved and assessed for eligibility. Ten percent of the full texts were screened by DG, and the agreement rate and Cohen’s kappa at this stage were 92.3% and 0.75, respectively. The final sample constituted 36 articles, comprising 33 articles that met the eligibility criteria as well as three articles obtained through screening the references of eligible articles and relevant publications [16,28]. See more details in Fig 1 and Table 1.

**Table 1:** Summary of studies included in the review.

Study	Sample and country	Birth year	Sample size (% Male)	Exposure (measure) *	Exposure age (years)^&^	Outcome (measure) ^#^	Outcome age (years)^&^
Berg et al. (2018)[46]	Ninth-grade pupils attending comprehensive school in Tampere, Finland	1967	2194 (NR)	Psychological symptoms (a checklist of 17 physical and psychological complaints)	15.9 (SD 0.3)	Frequency of intoxication (3 categories)	22
Kendler et al. (2018)[47]	All birth due between April 1, 1991 and December 31, 1992 in the Avon district, UK	1991~1992	7168 (49.1%)	Ext (sum score of antisocial behaviours)	13.5 and 15.5	Heavy episodic use weekly (AUIDT, binary)	20
						Alcohol problems (AUDIT, binary)	
Soloski et al. (2018)[48]	National representative sample of high-school adolescents (Add Health), USA	1976~1983	9330 (45.2%)	Depression (6 questions assessing depressive symptoms)	14.9 (11~18)	Binge Drinking (Days of 5+ drinks in a row over past 12 months, 6 categories)	21.6 (18~26)
Hoyland et al. (2017)[49]	National representative sample of high-school adolescents (Add Health), USA	1976~1983	2610 (44.5%)	Depression (9 items from CES-D scale)	15.6 (11~18)	Derived latent classes (ever had a drink, drinking frequency, drunk frequency, binge drinking frequency, drinking quantity, negative consequences)	29.6 (24–32)
Squeglia et al. (2017)[50]	Selective sample of students from local middle school, USA	NR	137 (56%)	Conduct disorder (DSM-IV)	12~14	Alcohol initiation	18 (16~19)
Edwards et al. (2016)[51]	All birth due between April 1, 1991 and December 31, 1992 in the Avon district, UK	1991~1992	4534~ 6598 (NR)	Conduct problems (SDQ)	11.8	Alcohol problems (20 questions from AUDIT, DSM-IV, and other negative consequences)	20
				Conduct problems (22 types of delinquent or anti-social behaviour)	15.6		
				Major Depression (SMFQ)	16.6		
Quinn et al. (2016)[52]	Nine-year-old twins identified in the Swedish Twin Registry, Sweden	1992~1995	15602 (51%)	Conduct problems (SDQ)	15	Alcohol problems (AUDIT)	18
Savage et al. (2016)[53]	Twins born from 1983 to 1987 in FinTwin12 study, Finland	1983~1987	1906–51.20%	Social anxiety (MPNI)	12	Frequency of drinking alcohol	22
Swift et al. (2016)[54]	Representative sample of the Victorian population of school pupils, Australia	1977~1979	1268–50.90%	Antisocial behaviour (self-report early delinquency scale)	Onset of antisocial behaviour from age 14/15 to 17	AUD (DSM-V)	24
				Anxiety and depression (revised CIS)	Persistence of depression /anxiety from age 14/15 to 17		
Thompson et al. (2016)[55]	Youth recruited by random digit dialing from a medium-sized Canadian city, Canada	1985~1991	622–49%	EXT (DSM-IV)	16~17	Frequency of 5+ drinks (binary)	18~19
				INT (DSM-IV)		Alcohol related harm (six items from the Harmful Effects of Alcohol Scale)	
Cook et al. (2015)[56]	National representative sample of high-school adolescents (Add Health), USA	1976~1983	5422–46.10%	Latent class of antisocial behaviour across time (adapted Health Behaviour Questionnaire)	Baseline 13.96 (SD 1.06), follow-up one year later	Problematic alcohol use (6-item alcohol related problems scale)	20.32 (SD 1.09)
Jun et al. (2015)[57]	Community-based sample from 80 neighborhood clusters, USA	1982~1985	724–51.00%	EXT (YSR) INT (YSR)	15	Drink or not in the past month	18
Pesola et al. (2015)[58]	All birth due between April 1, 1991 and December 31, 1992 in the Avon district, UK	1991~1992	2964–36%	Depression (SMFQ)	13.9 (SD 0.21)	Harmful drinking (AUDIT)	18.7 (SD 0.49)
Virtanen et al.(2015)[59]	All pupils who attended the last year of compulsory school (age 16) in all nine schools in a middle-sized municipality in Northern Sweden	1965	1001–51.80%	Depression (DSM-V) Anxiety (DSM-V)	16	Trajectory of average alcohol intake (multiply drinking frequency with drinking quantity per occasion)	5 waves from 16~45
Edwards et al. (2014)[60]	All birth due between April 1, 1991 and December 31, 1992 in the Avon district, UK	1991~1992	1637–37.80%	Trajectory of depression (SMFQ)	4 waves from 12~17	Binary harmful drinking (AUDIT) Latent alcohol use (AUDIT)	18.5
Kretschmer et al. (2014)[61]	All birth due between April 1, 1991 and December 31, 1992 in the Avon district, UK	1991~1992	7218 (NR, 52% in initial sample)	Trajectory of conduct problems (SDQ)	6 waves from age 4 to age 13	Binary harmful drinking (AUDIT)	17.9 (IQR17.7~17.11)
Pesola et al. (2014)[62]	All birth due between April 1, 1991 and December 31, 1992 in the Avon district, UK	1991~1992	3710–44%	Depression (SMFQ)	16	Alcohol problems (AUDIT)	18
Stanley et al. (2014)[63]	Community sample of urban Indian youths in the Seattle area, USA	1976~1978	281 (~48.3%)	EXT(CBCL) INT (CBCL)	11.7 (11~12)	AUD (DSM-IV)	19.7
Meier et al. (2013)[64]	Birth cohort of consecutive births between April 1, 1972, and March 31, 1973, in Dunedin, New Zealand	1972~1973	957 (~52%)	EXT (DSM-IV)	Average of 4 waves at age 5,7,9,11 Onset at age 11,13,15,18	AUD (DSM-IV)	3 waves from age 18 to age 32
				Anxiety (DSM-III)	Onset at age 11,13,15,18		
				Depression (DSM-III)	Onset at age 11,13,15,18		
				INT (RBQ)	Average of 4 waves at age 5,7,9,11		
Naicker et al. (2013)[65]	A representative sample of general population randomly selected by stratified two-stage design, Canada	1977~1983	1027–53.80%	Depression (Short Form for Major Depression)	12~17 at baseline, depression assessed at age 16~17	Heavy drinking (consumption of >16 drinks/wk for males and >11 drinks/wk for females), and/or consuming 5+ drinks in one sitting at a frequency greater than once a month)	Measured every two years from age 18/19 to 26/27
Green et al. (2012)[66]	Essentially all first grade students of Urban African Americans in the Woodlawn community area of Chicago, USA	1959~1960	1242–48.80%	Psychological distress (How I feel scale on anxiety and depression)	15~16	Drinking quantity when they were drinking the most in last year	32~33
McKenzie et al. (2011)[67]	Two-stage cluster sample selecting random class from 44 secondary schools in the state of Victoria, Australia	~1977	1758 (NR)	Number of waves when depression and anxiety symptoms over a threshold (revised CIS)	5 waves from age 15.5 to age 17.4	AUD (Composite International Diagnostic Interview (CIDI))	24
Stumm et al. (2011)[68]	Primary school students aged 6 to 12 in Aberdeen, Scotland in 1962, UK	1950~1956	12500 (~52.3%)	EXT (RBQ) INT (RBQ)	9.7 (SD 1.5)	Frequency of alcohol consumption, weekly alcohol units (category), number of hangovers last year and how often they consumed 4+ drink per occasion (category)	46~51
Bor et al. (2010)[69]	Pregnant women attending clinic visit at one hospital in Brisbane, Australia	1981~1984	3173 (~51.9%)	Anti-social behaviour (CBCL)	2waves at age 5 and age 14	Binge drinking (non-drinkers, 1~6 drinks, 6+drinking per occasion)	21
Hill et al. (2010)[24]	Youths recruited from 18 elementary schools in urban Seattle, USA	1975	640 (NR)	EXT (5 items, “How many times have you done the following things?” Done what feels good, no matter what?; Gone to a wild, out-of-control party?; Upset or annoyed adults just for the fun of it?; Done something dangerous because someone dared you to do it?; Done crazy things even if they are a little dangerous?	Average score at age 14 and15	AUD (DSM-IV)	27
				Anxiety (CBCL)			
Huurre et al. (2010)[70]	Ninth-grade pupils attending comprehensive school in Tampere, Finland	~1967	1387–44.20%	Depression (seven items indicative of depression (lack of energy; sleeping difficulties; nightmares; fatigue; irritability; loss of appetite; and nervousness/anxiety))	15.9 (SD 0.3)	Excessive alcohol use (AUDIT)	32
Colman et al. (2009)[71]	A stratified sample of every child born in England, Scotland, or Wales during one week in March 1946, UK	1946	3652–51.90%	EXT (RBQ)	2 waves at age 13 and 15	Alcohol abuse (CAGE) (number of waves with alcohol abuse)	2 waves at age 43 & 53
Maggs et al. (2008)[21]	all children born in Great Britain between 3 and 9 March 1958, UK	1958	4758~12772 (~50.8%)	EXT (RBQ) INT (RBQ)	At age 7 and 11	Weekly alcohol units Harmful drinking (CAGE)	At age 23, 33, 42
Pitkanen et al. (2008)[72]	Twelve complete (the initial participation level was 100%) school classes of second-grade pupils in the town of Jyväskylä, Finland	1959	347–53.00%	Anxiety (easily starts crying if others treat him/her nastily, afraid of other children; and cries easily at age 8; fearful and helpless in other’s company, target of teasing, unable to defend at age 14)	At age 8 and 14	Heavy drinking (police records, annual drinking etc.) by age 20 (4 categories) Annual frequency of drinking at age 27, 42 (days) Frequency of binge drinking at 27, 42 (6 categories) Harmful drinking (CAGE score) Problem drinking by 27, by 42 (whether experienced any difficulties, 6 categories)	At age 20, 27, 42
Timmermans et al. (2008)[73]	Randomly from the Dutch province of Zuid Holland, using inoculation registers and the municipal population register of Rotterdam in 1989, Netherland	1986~1987	309–48.90%	Trajectory of EXT (CBCL)	3 waves at 4/5, 10/11, 18	Alcohol use (combination of drinking frequency and drunkenness, 7 categories)	18.19 (SD 0.7)
Pardini et al. (2007)[74]	Randomly selected from a list of names and addresses of all seventh-grade boys in participating Pittsburgh public schools during 1987–1988, USA	~1973	506–100%	Conduct disorder (DSM3, SRD, YSR)	13.9 SD NR	AUD symptoms, AUD onset (DSM III/IV)	20.4–25.4
				Anxiety (YSR, Teacher Report Form and CBCL)			
				Depression (Recent Moods and Feeling Questionnaire)			
Niemela et al. (2006)[23]	10% of all birth born in 1981, a representative sample of communities, Finland	1981	1967–100%	EXT (RBQ)	8	Frequency of drunkenness (4 categories)	18
				INT (RBQ)			
Moffit et al. (2002)[75]	Consecutive births between April 1972 and March 1973 in Dunedin, New Zealand	1972~1973	457–100%	Antisocial behaviour (RBQ/SRD)	6 waves at age 5, 7, 9, 11, 15, 18	AUD (DSM-IV)	26
Moffitt et al. (1996)[76]	Consecutive births between April 1972 and March 1973 in Dunedin New Zealand	1972~1973	457–100%	Antisocial behaviour (RBQ/SRD)	6 waves at age 5, 7, 9, 11, 15, 18	AUD (DSM-III)	18
Steele et al. (1995)[77]	An urban community sample of Caucasian adolescents in the southeastern region, USA	NR	187–47.10%	Conduct problems (Revised Behaviour Problem Checklist)	13.5 (11.1–15.8)	Potential alcohol dependence (MAST)	19.75 (17.8,22.4)
				Anxiety (Revised Behaviour Problem Checklist)			
Pulkkinen et al.(1994) [78]	Second-grade pupils (8~9 years old) in the town of Jyvaskyla, Finland	1959~1960	369–53.10%	Conduct problems (teacher ratings on punishments at school, truancy, smoking, drinking and contacts with the police)	14	Problematic drinking (CAGE)	26–27
				Anxiety (Peer nomination, "Who is fearful, helpless in others' company, a target of teasing, unable to defend himself or herself?)			

*EXT: externalising problems; INT: internalising problems; CES-D: Center for Epidemiologic Studies—Depression Scale; SDQ: Strengths and Difficulties Questionnaire; SMFQ: Short Mood and Feelings Questionnaire; MPNI: Multidimensional Peer Nomination Inventory; YSR: Youth Self-report scale; RBQ: Rutter Behaviour Questionnaire; CBCL: Childhood Behaviour Check List; SRD: Self-Reported Delinquency Scale.

^&^ SD: standard deviation; NR: not reported; IQR: interquartile range.

^#^AUD: alcohol use disorder; CIS: Clinical Interview Schedule; AUDIT: Alcohol Use Disorder Identification Test; CAGE: cut-down, annoyed, guilty, eye-open scale; DSM: diagnostic and statistical manual; MAST: Michigan Alcohol Screening Test; NR: not reported.

An extraction form was developed by KN, and 10% of the selected articles were extracted by DG. The information extracted included author, year of publication, country (proxy for culture) and sampling strategy, sample size (proportion of male) and their birth year (proxy for history), measurement scale of exposure and outcome, age when exposure and outcome were measured, direction and size of the association, sex differences of the association, covariates adjusted for, statistical models, assessment of attrition bias, and methods for dealing with missing data. Associations were extracted if they reflected the total association of the relationship. For example, if depression at age 7 and depression at age 16 were adjusted for in the model simultaneously (e.g. outcome = a+b1*depression at age 7+b2*depression at age 16), then the coefficient b2 can be interpreted as total association between depression at age 16 and alcohol, which was not confounded by previous depression status (and thus was extracted), while the coefficient b1 was the controlled direct association between depression at age 7 and the outcome not through later depression status (and thus was not extracted). See more definitions in Pearl(2001) [79]. In addition, some studies reported several associations for the same exposure-outcome set, so other rules were devised to avoid duplicate associations: continuous measures (versus categorical), self-report (versus parent or teacher report), most properly adjusted result, unstandardized betas (versus standardised), whole population (versus sub-population). Discrepancies were discussed and agreed upon before extracting information from all included articles.

### Data synthesis

As shown previously [16], there was high heterogeneity in the studies included in this review in terms of sample characteristics (a wide age range of participants), subtype and developmental timing of mental health problems and alcohol use behaviours (binary/continuous, trajectory/one-time-point, measurement scales), length of follow-up, and confounders adjusted for. Approaches exist to overcome the heterogeneity due to analytical approaches [80], with the exception of exposure heterogeneity. Particularly, for continuous exposures, it was not possible to standardise or transform them to the same metric required by meta-analysis [81]. In addition, the number of articles is too low to be pooled after taking into account the potential factors examined in our study [82]. We, therefore, report results narratively, and present extracted associations in detail in S2 Table.

**Fig 2:**
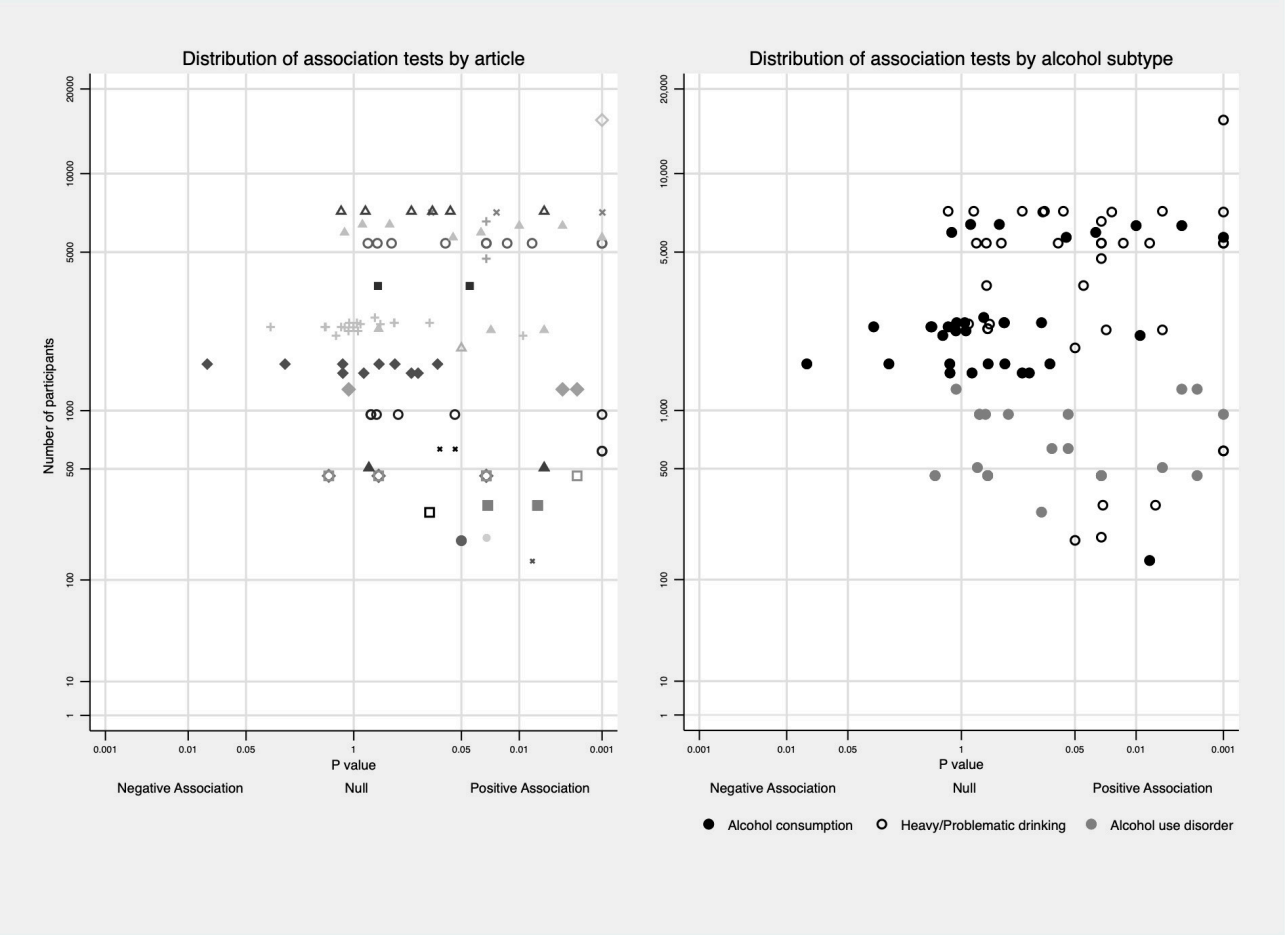
Distribution of association tests between EXT and subtypes of alcohol use behaviours. Distribution of association tests clustering in one article was plotted on the left to show the non-independence among tests.

**Fig 3:**
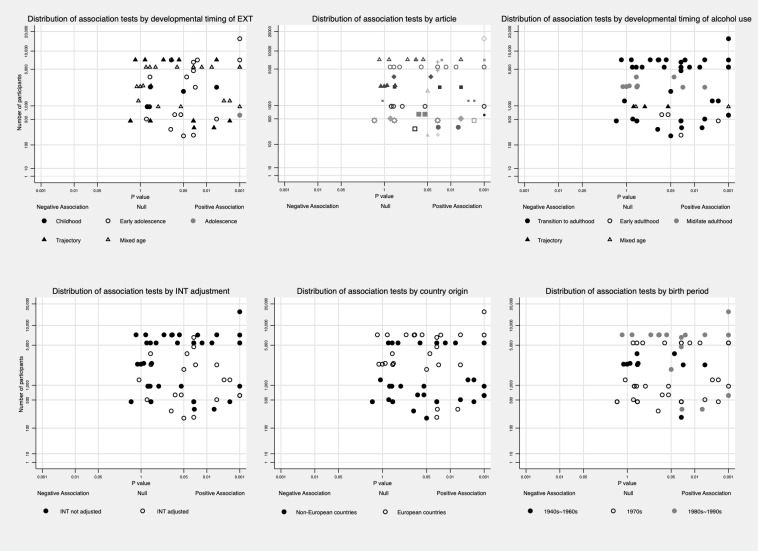
Distribution of association tests between EXT and alcohol problems among various subgroups. Association tests were limited to those using heavy/problematic drinking, AUD as the outcome, and distribution of association tests nested in one article was plotted in the upper middle for easy comparison.

**Fig 4:**
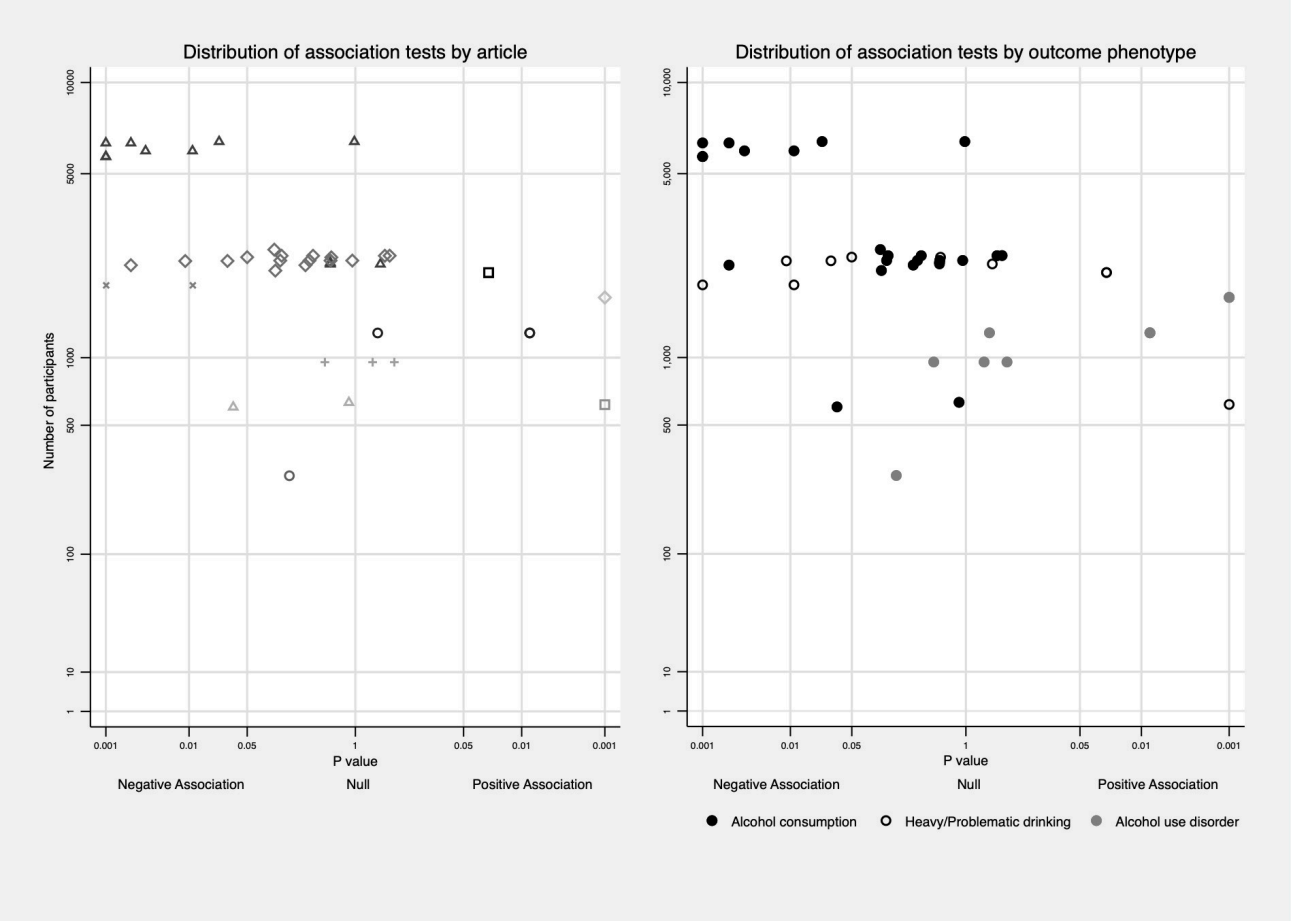
Distribution of association tests between INT and subtypes of alcohol use behaviours. Distribution of association tests clustering in one article was plotted on the left to show the non-independence among tests.

**Fig 5:**
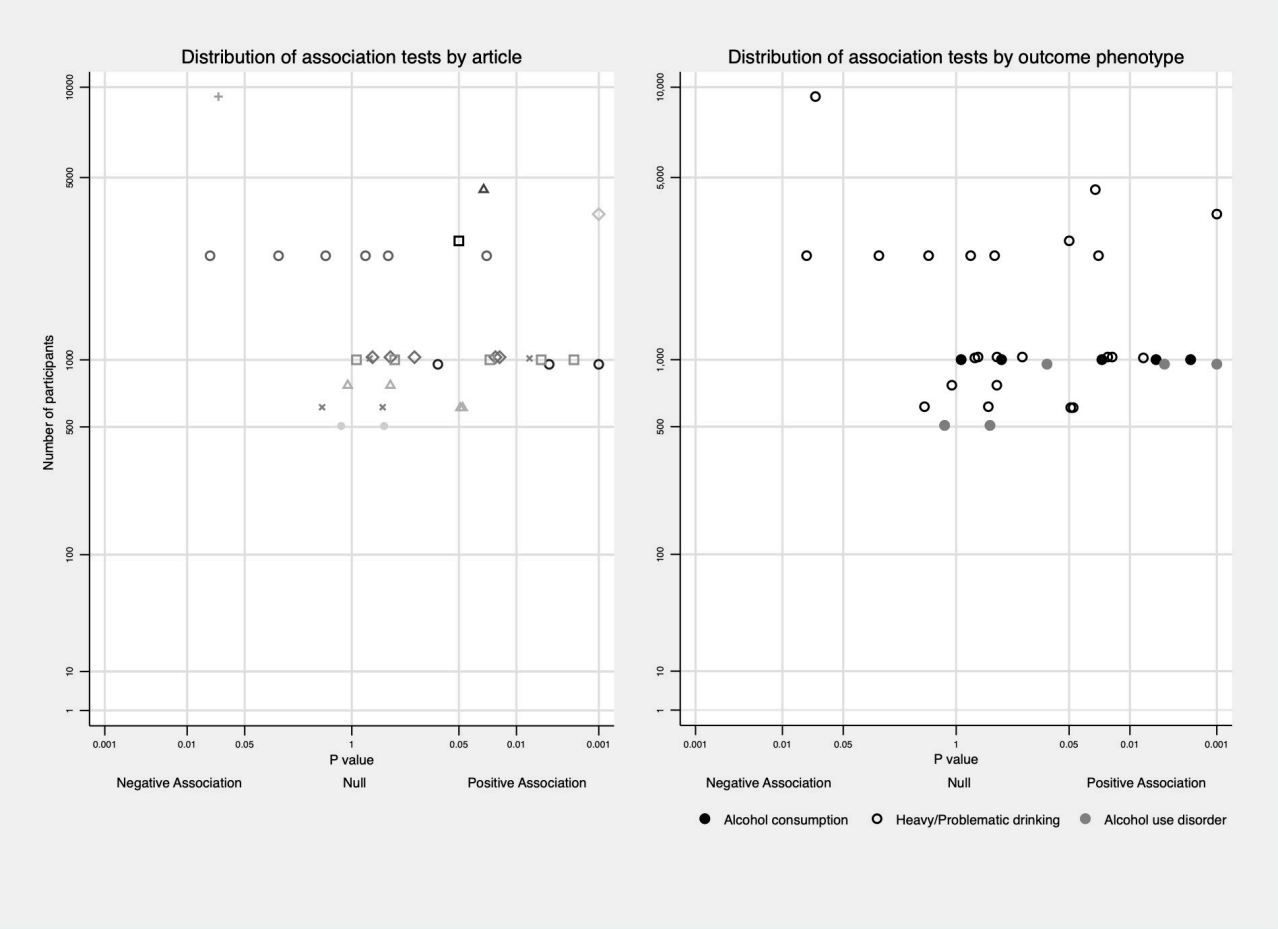
Distribution of association tests between depression and subtypes of alcohol use behaviours. Distribution of association tests clustering in one article was plotted on the left to show the non-independence among tests.

**Fig 6:**
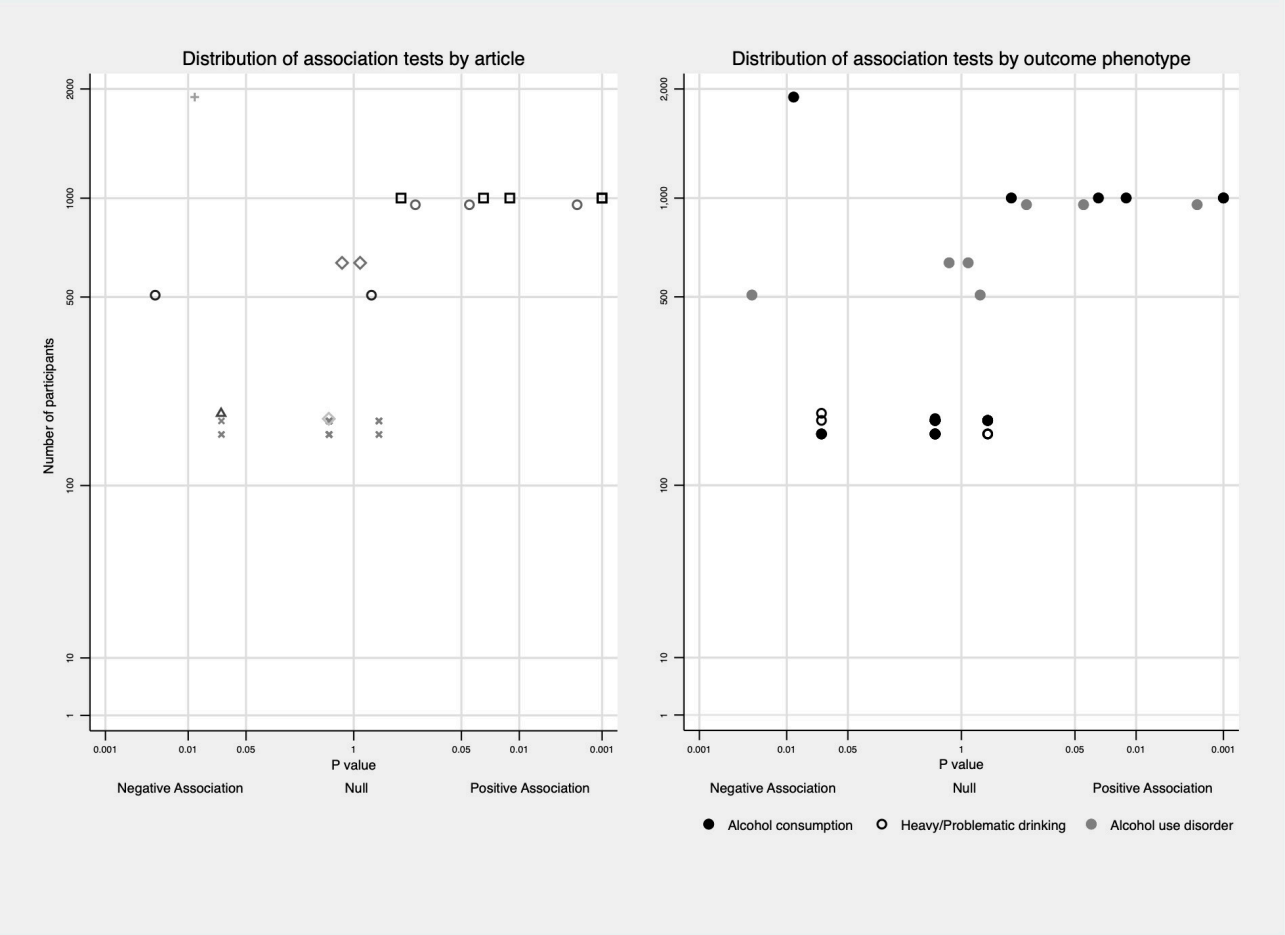
Distribution of association tests between anxiety and subtypes of alcohol use behaviours. Distribution of association tests clustering in one article was plotted on the left to show the non-independence among tests.

To minimize potential bias caused by different ways of reporting the results (e.g. different exposure and outcome categories, reporting separately by sex/age), or by articles using the same population, data were synthesised in the following three ways: a) We reported the proportion of tests that were significant (*P* value equal or less than 0.05) out of all tests that reported specific exposure-outcome sets regardless of studies/articles as did by Hussong et al. [16]. For example, for the INT and alcohol consumption set, article A reported 4 tests of this association (mild INT vs. no INT in males, serve INT vs. no INT in males, mild INT vs. no INT in females, severe INT vs. no INT in females), and only association for serve INT vs. no INT in males was significant negative; article B reported 2 tests (one in males, one in females), and neither of them was significant. Then, the proportion of negative association would be 1 / (4+2) = 16.7%. b) We reported the proportion of studies that reported significant association for each exposure-outcome set. For each study (using the same dataset) and for each exposure and outcome pair, no matter how many tests were reported, and the association was counted as significant as long as one test was significant. For instance, in the example above, article A would be counted as reporting a significant negative association between INT and alcohol consumption, and article B would be counted as reporting no association between INT and alcohol consumption. Then the proportion would be 1 / (1+1) = 50%. c) The method outlined in point a) was repeated in high-quality studies as defined below. We report results synthesised using the first method in the main article and other results in S3 and S4 Tables for readers’ information. To maximize the use of available information and informed by the albatross plot [83], we also described the distribution of *P*-values for each association test against its sample size in Figs 2–6. In addition, average *P*-value across subtypes and developmental timing of both exposure and outcome was presented in S1–S4 Figs.

### Quality assessment

We used an adapted version of the Critical Appraisal Skills Programme (CASP) Cohort Study Checklist [84], which was shortened to 8 questions, as shown in S5 Table. We mainly assessed four aspects of each cohort study: sample selection, measurement error, core confounder adjustment, and the handling of missing data, which are key issues that can cause bias in observational studies [85,86].

A quality score (QS) was assigned for each question. The scores were then summed, with total scores ranging from 0 to 8. Studies with scores ranging from 0–4 were considered as poor quality, and studies with score 5–8 as good quality. Quality assessment was done for all 36 selected articles by two researchers separately and disagreements were discussed to reach a final consensus.

We organise the results by four subtypes of mental health problems: EXT, INT, depression and anxiety. Within each domain, we further structure our findings for three subtypes of alcohol use behaviours. Where appropriate, we further explored whether the results were affected by whether adjusting for EXT and INT accordingly, the developmental timing of exposure and outcome, and country origin or birth cohort; we also summarised the evidence for potential sex differences.

## Results

### Search results

Of the 36 articles included in this review, eleven studies were carried out in the US and nine in the UK, followed by six in Finland. The data used were from over 20 longitudinal studies, but six articles used data from the Avon Longitudinal Study of Parents and Children (ALSPAC). The sample size in 22 articles was over 1000.

Fifteen of the 36 articles were rated as high quality due to their large sample size, representativeness of the population, inclusion of core confounding factors and advanced principles to deal with missing data [21,24,46–48,51,54,56,58,60,62,63,66,67,74]. By comparison, 21 of the 36 studies were rated as poor quality due to their failure to control for potential confounding factors, small sample sizes, and improper strategies for missing data (mainly complete case analysis) [23,49,50,52,53,55,57,59,61,64,65,68–71,73,75–78]. We summarised the frequency of the potential confounders that were adjusted for in the 36 articles in S6 Table to give a more comprehensive picture.

Twelve out of 36 articles focused explicitly on the internalising domain as the exposure, 9 articles focused on the externalising domain, and 14 articles explored both domains. This resulted in 26 articles on the internalising domain (INT: n = 9, depression: n = 13, anxiety: n = 8) and 23 articles on the externalising domain. With regard to alcohol use behaviours, the distribution was as following (more details in S1 Text): alcohol consumption (n = 9) [21,50,53,57,59,66,68,69,72], heavy/problematic drinking (n = 22) [21,23,46–49,51,52,55,56,58,60–62,65,68,70–73,77,78] and AUD (n = 8) [24,54,63,64,67,74,75,87].

Most articles measured mental health problems at one time point: four in childhood [21,23,64,72], 17 in early adolescence, seven in adolescence [51,55,59,62,65,67,70], and 6 with wide age range [48,49,54,64,66,68]. Six articles derived mental health trajectories as an exposure [60,61,69,73,75,76]. Alcohol use behaviours was measured during early adulthood in 26 articles, mid-adulthood in eight articles [21,24,65,66,70,72,75,78], late adulthood in four articles [21,68,71,72] and modelled as trajectories across adulthood in two articles [59,64,65].

### Association between externalising problems and alcohol use behaviours

Our review identified 103 tests of the association between EXT and alcohol use behaviours in 23 articles, and higher early life EXT was significantly associated with more alcohol-related issues later in 37 tests (35.9%).

With respect to the association between EXT and different alcohol subtypes, a higher number of positive associations were found with more severe alcohol outcomes (See the distribution of *P*-value across subtypes of alcohol use behaviours in Fig 2). Unique associations between EXT and alcohol consumption were examined in 37 tests [21,50,57,68,69]. Six (16.2%) tests reported positive associations, one test found negative association, and the rest reported no association. 42 tests in 13 articles examined heavy/problematic drinking as an outcome [21,23,47,51,52,55,56,61,68,71,73,77,78], and 22 (52.4%) tests reported positive associations. For AUD, 24 tests in seven articles [24,63,64,74,88,89] were extracted, and nine (37.5%) tests reported positive associations. Results are presented in Table 2.

**Table 2:** Distribution of associations across subtypes of mental health problems and alcohol use behaviours.

	Alcohol consumption			Heavy/problematic drinking			Alcohol use disorder		
	positive	negative	no	positive	negative	no	positive	negative	no
Externalizing domain	6/37 16.2%	1/37 2.7%	30/37 81.1%	22/42 52.4%	0/42 0%	20/40 47.6%	9/24 37.5%	0/24 0%	15/24 62.5%
Internalizing domain									
Internalizing	0/26 0%	9/26 34.6%	17/26 65.4%	3/14 21.4%	5/14 35.7%	6/14 42.9%	4/9 44.4%	0/9 0%	5/9 55.6%
Depression	3/5 60%	0/5 0%	2/5 40%	9/24 37.5%	2/24 8.3%	13/24 54.2%	2/5 40%	0/5 0%	3/5 60%
Anxiety	4/13 30.8%	2/13 15.4%	7/13 53.8%	0/31 0%	2/31 6.4%	29/31 93.6%	2/7 28.6%	1/7 14.3%	4/7 57.1%

Then, we explored the variation of association according to the developmental timing excluding alcohol consumption outcomes. Two out of seven (28.6%) tests measuring EXT in childhood reported positive associations with later alcohol use behaviours [21,23,64], 10 out of 19 (52.6%) tests measuring EXT in early adolescence showed positive associations [21,24,47,51,52,56,63,71,74,77,78], and two tests measuring EXT in adolescence both reported positive associations [55]. Results from four papers using EXT trajectories indicated that EXT in adolescence might be more strongly related to alcohol outcomes, especially the persistence of EXT from childhood to adolescence [61,73,75,76] with the exception of Bor et al.’s study [9]. This association pattern was also reflected in papers that measured EXT at several time points [47,51,64] with the exception of Maggs et al.’s study [21]. Out of 41 tests measuring alcohol use in transition to adulthood, 23 (56.1%) presented positive associations between EXT and alcohol outcomes [23,47,51,52,54–56,61,63,73,74,76,77]. Four out of nine (44.4%) tests measuring alcohol use in early adulthood presented positive associations; however, alcohol use was measured at around 26/27 years old in these studies [24,75,78]. By comparison, three out of ten tests (30%) measuring alcohol use in midlife and above showed positive associations [21,68,71]. Besides, 13 out of 23 (56.5%) tests adjusting for INT simultaneously reported positive associations, while 18 out of 43 (41.9%) tests not adjusting for INT reported positive associations. More information is in Fig 3.

Among the 23 articles, 19 included both males and females in their samples, and four explored associations only among males [23,74–76]. Eleven of the 19 articles that included both sexes did not explore whether there was an interaction between sex and EXT in the association with later alcohol use [21,24,47,50,52,54,61,63,64,68,69]. Only two of the remaining articles reported a significant sex interaction with the association being stronger in males [56], while the other six articles found no statistically significant interaction [51,55,57,71,73,77]. Among tests reporting the association separately in male and female, 15 out of 42 (35.7%) in male were significantly positive; 6 out of 28 (21.4%) in female were significantly positive. To explore the role of culture and history, we categorized country origin into two groups (Europe versus non-Europe), and birth year into three cohorts (born in or before 1960s, born in 1970s, born in or after 1980s). Proportion of positive results were similar across continents (Europe 44.8% versus non-Europe 48.6%) (See Fig 3); four out of twelve tests (33.3%) among those born in or before 1960s reported positive associations, 15 out of 34 tests (44.1%) among those born in 1970s reported positive associations, and 11 out of 19 tests (57.9%) among those born in or after 1980s reported positive associations (See Fig 3). To tease out age effect from cohort effect, analysis was further limited to those born in 1970s and had their alcohol measured during transition to adulthood, 11 out 21 tests (52.4%) found positive results.

#### Summary

EXT was positively associated with later alcohol use and this association varies across subtypes of alcohol use behaviours: higher proportion of positive associations for more severe outcomes. More positive associations were detected when EXT was measured in adolescence and alcohol use in transition to adulthood. The probability of detecting significant positive associations between EXT and later alcohol use behaviours was higher when adjusting for INT simultaneously. Most of the studies that tested sex effect in the association detected no significant interaction, however, higher proportion of positive results were reported in male population. The probability of detecting a positive association between EXT and alcohol use behaviours appeared to be consistent across countries and cohorts.

### Association between the internalising domain and alcohol use behaviours

In the26 articles exploring the association between the internalising domain and alcohol use behaviours, 135 tests were extracted, including 49 tests in 11 articles investigating INT as the exposure [21,23,46,54,55,57,63,64,66–68], 34 tests in 11 articles using depression [48,49,51,58–60,62,64,65,70,74], and 52 tests in 8 articles assessing anxiety [24,53,59,64,72,74,77,78].

#### Internalising problems and alcohol use

Among 49 tests investigating the association between INT and alcohol use behaviours, seven tests found a positive association [46,54,55,67], 14 produced a negative association [21,23,66,68], and 28 found no association.

With respect to subtypes of alcohol use behaviours (See Fig 4), 9 out of 26 (34.6%) tests found negative associations with alcohol consumption [21,66,68] while the rest reported no association [21,57,66,68]. 14 tests in five articles used heavy/problematic drinking as an outcome; three (21.4%) of them found a positive association [46,55], and five (35.7%) tests indicated negative association [23,68]. Out of nine tests in four articles with AUD as an outcome, four (44.4%) tests reported a positive association [54,67] and 5 found no association [63,64].

Due to the divergent direction of the association, analysis was done with respect to each subtype of alcohol outcome and summarized as following: for alcohol consumption, higher proportion of negative associations was detected when EXT were simultaneously adjusted for (7 out of 8 tests vs. 5 out of 27 tests); for heavy/problematic drinking, it was more likely to detect positive associations when INT were measured at adolescence; for AUD, which was mainly measured during transition to adulthood, significant positive associations were reported when INT were measured during early adolescence and adolescence and when EXT were simultaneously adjusted for. There was no country-source heterogeneity within subtype of alcohol outcomes, but none of the studies using AUD as an outcome was from European countries. Cohort effect cannot be explored as the majority of the tests on alcohol consumption were from participants born in or before 1960s, and all tests on AUD were from participants born in 1970s.

#### Depression and alcohol use

14 out of 34 (41.2%) tests using depression as the exposure showed positive associations [49,51,58–60,62,64,65,70], while three tests showed negative associations [48,49] and 17 tests showed no association. Within sub-categories of alcohol use behaviours (more information in Fig 5), three out of five tests reported a positive association between depression and alcohol consumption [59]; among 24 tests in eight articles using heavy/problematic drinking as an outcome [48,49,51,58,60,62,65,70], nine (37.5%) tests reported a positive association [49,51,58,60,62,65,70] and three (12.5%) presented negative associations [48,49]; two out of five (40%) tests indicated positive associations between depression and alcohol use disorder [64] and no association was found by the remaining tests [64,74].

No further exploration was carried out due to the limited number of tests for alcohol consumption and AUD. With respect to heavy/problematic drinking, among 12 tests that adjusted for EXT, four (33.3%) tests reported positive associations while two (16.7%) tests reported negative associations; among 12 tests that did not adjust for EXT, five (41.7%) found positive associations while one (8.3%) found negative associations. No conclusion can be drawn regarding the development period for depression as depression was mainly measured during adolescence. As for country and cohort differences, negative associations were only detected in one national study in the USA [48,49].

#### Anxiety and alcohol use

We identified 52 tests for the association between anxiety and alcohol use, one of which measured social anxiety [53]. Six out of 51 tests measuring general anxiety indicated positive associations [59,64] and five produced negative associations [72,74,78]. Negative association between social anxiety and alcohol consumption was reported [53].

For alcohol consumption, two out of thirteen tests in one article showed negative associations [72] and four tests found positive associations [59]. For heavy/problematic drinking, two out of 31 tests in two articles showed negative association [72,78], and no statistically significant association was detected for the remaining tests [72,77]. Out of seven tests identified from three articles with alcohol use disorder as an outcome [24,64,74], two tests found positive associations [64] and one test reported negative associations [74]. The distribution of *P*-value against sample size for anxiety and alcohol use behaviours is shown in Fig 6, and no systematic pattern of the association can be observed.

It should be noted that when anxiety was measured during early adolescence, only negative associations were found [72,74], while positive associations were only reported when anxiety was measured during adolescence [64,90]. No significant associations were reported when anxiety was measured during childhood. Two out of seven tests that adjusted for EXT reported negative associations, while three out of 44 tests found negative associations and six out of 44 tests reported positive associations when EXT were not adjusted for. No exploration for country or cohort effect can be done after taking into account the influence of developmental timing of anxiety.

#### Summary

Evidence for the association between internalising domain and alcohol use behaviours were inconsistent but somewhat varied across subtypes of the internalising domain and alcohol use behaviours. The relationship between INT and alcohol use behaviours tended to be negative for mild alcohol behaviour, especially when EXT was adjusted for, and positive for severe alcohol outcomes. The association between depression and alcohol outcomes seemed to be positive across subtypes. The association between anxiety and alcohol use behaviour was equivocal, and the reason might be that anxiety at different developmental timing was associated with later alcohol use behaviours in a different way.

24 out of 26 articles about the internalising domain had both males and females in their studies, and 11 of them did not explore sex differences in the associations between internalising domain and alcohol outcomes [21,24,49,54,58,63–65,67,68,70]. Among the 13 studies that explored sex differences, three articles found significant sex differences [60,66,78], while the remaining ten articles reported no sex differences [46,48,51,53,55,57,62,72,77,91]. More studies are needed to draw conclusion on the potential influence of country and cohort on the association between internalising domain and alcohol use behaviours.

### Sensitivity analysis

For EXT, 19 out of 36 (52.8%) tests from high-quality studies reported positive associations with alcohol use behaviours [21,24,47,51,63,74,88,89,91], while 18 out of 67 (26.9%) tests from poor-quality studies reported positive associations [23,50,52,61,64,68,71,73,77]. With respect to the internalising domain, ten out of 36 (27.8%) tests from good-quality studies reported positive associations [46,46,51,54,58,60,62,67] and ten tests (27.8%) reported negative associations [21,48,66,74], while 17 out of 99 (17.2%) outcomes from poor quality studies reported positive associations [49,49,55,59,64,65,70] and 12 out of 99 (12.1%) found negative associations [23,49,53,68,72,78].

Results synthesised with subtype of alcohol use behaviours among high-quality studies are presented in S4 Table. There are some discrepancies with our main results: the trend that the proportion of positive associations between EXT and alcohol use increases with the severity of the outcome became less obvious (alcohol consumption: 50%; heavy/problematic drinking: 60%; alcohol use disorder: 37.5%); only negative associations were found between INT and alcohol consumption and only positive associations were detected between INT and more severe alcohol outcomes (heavy/problematic drinking, alcohol use disorder); no significant association in either direction was found between depression and alcohol use disorder; only two high-quality studies examined the association between anxiety and AUD, and one of them reported negative association.

The analysis done by extracting one association item from studies using the same dataset did not change our conclusion drawn from our main results.

## Discussion

This systematic review investigated the association between early life mental health and alcohol use behaviours in adulthood. The evidence indicates positive associations between EXT and later alcohol use behaviours, but this association tends to vary with subtypes of alcohol use behaviours, with more severe outcomes being more consistently linked with EXT. EXT measured during early adolescence and adolescence appears to be more sensitive compared to that in childhood. The association between the internalising domain and alcohol use behaviours is inconclusive with both positive and negative associations presented for the same subtype of alcohol use behaviours.

### Association between externalising problems and alcohol use behaviours

Our review points to a positive association between early life EXT and various alcohol use behaviours with more consistent positive associations being observed as the outcome becomes more severe (from alcohol consumption to problem drinking to AUD). This trend can also be seen from the distribution of *P* values across subtypes in Fig 2. Publication bias may underlie this finding since papers reporting no/negative associations are less likely to be published, but this pattern can also be seen when looking at different alcohol use behaviours within one study [23,61,64,68,92].

Based on current evidence, EXT in early adolescence and adolescence seems to play a more important role than that in childhood. This seems to contradict the hypothesis of the critical period, which emphasises that aversive experiences in late childhood (age 8–11) are especially impactful on later substance use and other behavioural problems [93]. The hypothesis states that children at this stage start to form their own identity, which is the basis for later behaviours and decisions; at the same time, they start to build affiliations with their surroundings and can easily get involved with deviance-prone peers if they manifest conduct problems themselves. Results from studies which derived trajectories of EXT seem to support the notion of “cumulative continuity”. The hypothesis of cumulative continuity stresses that the continuity of EXT rather than their severity matters in the development of behavioural problems later in life [25,61,94]. Future study should focus on trajectories of EXT as an exposure to better articulate the hypothesis of critical period (adolescence versus childhood) and the notion of cumulative continuity.

### Association between internalising domain and alcohol use behaviours

Compared to EXT, the associations between the subtypes of internalising domain and alcohol use behaviours are less consistent. Inconsistency may arise from the co-occurrence of EXT, which were not adjusted for in more than half of the selected studies. The proportion of positive/negative association differed between tests adjusting for EXT and those not adjusting for it, especially for INT (negative association for alcohol consumption: 87.5% vs. 18.5%). More effort needs to be made to understand how INT and EXT operate in tandem in children’s lives to increase or decrease the risk for alcohol use/problems in adulthood, as EXT is quite prevalent across different levels of INT [25]. For example, a positive association between depression and AUD was found only in participants with high levels of conduct problems and not in those with low and moderate conduct problems [74]. Moreover, one small sample size study found that pure EXT (without INT) had the strongest positive association with adolescent alcohol use, but this association became weaker when EXT co-occurred with INT [27]. Meanwhile, pure INT (without EXT) presented a negative association, though it was statistically non-significant [27]. Colder et al. also found that the highest probability of alcohol use was observed in those with high EXT and low INT, and a negative association between INT and alcohol use was strongest for youth with no EXT [95].

However, although studies mentioned above consistently showed an interaction between the externalising and internalising domains, opposite associations with alcohol use were detected across subtypes of internalising domain (positive between depression and alcohol use [74] but negative between INT and alcohol use [27,95]), as is indicated by our review (See Figs 4–6). Further implication would be that heterogeneity in measurement tools/instruments may underlie these inconsistencies in the literature as well. In 36 articles, five measurement tools were used to assess EXT, whereas more than ten tools were used for the internalising domain. It may be the case that these various tools measure different aspects of INT that exhibit different associations with alcohol use behaviours. A good illustration would be the differences between general anxiety and social anxiety. Articles that used social anxiety as exposure found negative associations with later alcohol use behaviours [53,96,97], while articles measuring general anxiety but using a scale that tended to reflect symptoms of social anxiety (“too dependent on adults,” “afraid of going to school,” “self-conscious or easily embarrassed,” “shy or timid,” “keeps from getting involved with others” [74], “fearful and helpless in other’s company, target of teasing, unable to defend” [72]) also reported negative associations. Thus, it could be argued that these scales measure different aspects of anxiety, and consequently, the effect of these aspects of anxiety on alcohol behaviour may differ. For example, a person who has social anxiety might be at lower risk for getting involved with alcohol because he/she may be less exposed to other adolescents who drink, or may not have the skills to obtain alcohol if he/she is below legal drinking age [53]; however, a person with other types of anxiety may have a higher risk for later alcohol use [96].

Another finding worth our attention is how the direction of the association between internalising domain and alcohol use behaviours flipped across the subtypes of alcohol outcomes, especially for INT. One possible explanation for this might be the U or J shaped association reported in cross-sectional studies [98–100]. Studies that reported negative association between INT and alcohol consumption were either large sample-size studies or measured alcohol consumption in mid-adulthood [21,66,68]. Under this situation, the majority of the participants would be non-drinkers or light drinkers, and negative association would be found when the relationship was modelled as linear. By comparison, for more severe outcomes, which were mainly measured at transition to adulthood [46,54,55,63,67] when alcohol use reached its peak, the results may reflect the positive association. Interestingly, a U-shaped pattern was also observed in a recent prospective study, which discovered that adolescents with more symptoms of depression were more likely to be either abstainers or to demonstrate a problematic use [49]. Researchers should take into account the potential non-linear relationship in the future.

Even though differences were observed in some studies between males and females [21,68], sex does not appear to be a substantial factor that caused the inconsistencies in our review. However, more attention should be paid to the role sex plays in the association between early mental health and later alcohol use behaviours due to the profound sex differences in the development of mental health, physiological vulnerability to alcohol, alcohol consumption patterns, and social norms and expectations about drinking [17]. No obvious country or history differences were discovered in our review after taking other factors into account. This may indicate that the association between early life mental health and alcohol use behaviours in adulthood reflect general developmental trends rather than specific historically bounded ones or culture specific ones [22]. However, studies comparing the historical differences or cross-countries comparison (especially in non-Western countries) are needed, as none of the studies included in our review tried to answer this question directly.

### Other implications for future studies

Several implications for future studies emerged from our review. Future work should examine whether the association between early life EXT/INT and alcohol use in adulthood can be interpreted as causal. Although causality in observational data is not easy to infer, a range of techniques such as cross-contextual comparisons, negative controls, sensitivity analysis for unmeasured confounders, instrumental variable analysis or Mendelian Randomization [101,102], and fixed-effect models that eliminate time-invariant confounders [103] can be used for more robust causal inference. To the best of our knowledge, only two articles in this area have applied fixed-effect models [104,105]. However, their exposure and outcome were measured within the same period and the direction of the association they found could be from alcohol use behaviours to mental health problems [104,106].

Moreover, the fact that almost half of the selected articles were rated as poor quality, and the fact that many high-quality studies did not account for missing data, raise more concern. Principled techniques to deal with missing data, such as inverse probability weighting, multiple imputations, full information maximum likelihood, or even combinations of these techniques [107–109] have been shown to return valid estimates under the missing at random assumption [110] and should be applied more often in the future.

### Strengths and limitations

This systematic review built on previous reviews that focused on alcohol use in adolescent [16] and extended AUD into adulthood. Also, we included both domains of mental health problems (EXT and INT) and subcategorised alcohol behaviours according to their level of severity, which provided new insights into these associations. Furthermore, we summarised evidence for a potential age effect and sex differences, although no conclusive findings can be drawn. Several limitations need to be considered when interpreting the results. First, based on current theory, this review focused on broad categories of mental health problems, which resulted in missing studies on Attention Deficit Hyperactivity Disorder (ADHD) and specific anxiety subtypes. Recent studies have shown that ADHD is also positively associated with later alcohol use [88,111], and it is very likely that a particular trait within the domain of the disorder is the driver for later alcohol use [112]. Future studies should compare how the associations may change when focusing on different symptoms within a certain disorder, such as aggression, impulsivity, sensation seeking under externalising domain, and social withdrawal under internalising domain. Second, though we tried alternative ways of data synthesis to avoid the risk of bias, we were not sure about the discrepancies discovered and only reported one set of the results in detail as Hussong et al. did [16]. Results using alternative data synthesis methods are attached in S3 and S4 Tables for the readers’ consideration. Third, due to the large number of articles retrieved (over 17,000), only a subset of the articles was reviewed by the second author. Forth, studies were restricted to articles published in English, and as a result, results may not be generalizable to other populations and may suffer from publication bias. However, we postulate that publication bias may exaggerate the proportion of positive associations for externalising problems to a limited extend and would not affect the results for internalising problems much, as the reported associations were already quite mixed.

## Conclusion

This review evaluated the evidence on the association between early life externalising/internalising problems and alcohol use behaviours in adulthood. For externalising problems, consistent positive associations were found across studies, and there tended to be more positive associations with more severe alcohol outcomes such as heavy/problematic drinking and AUD. Externalising problems in early adolescence and adolescence seem to be more strongly associated with alcohol outcomes than that in childhood. The evidence on associations between internalising problems and alcohol use behaviours is inconclusive, and the results suggested that different domains of internalising problems may differ in their associations with later alcohol use.

## Supporting information

S1 Checklist PRISMA 2009 checklist. (DOC)

S1 Table Key words for systematic review. (DOCX)

S2 Table Extracted associations for each exposure-outcome set in 36 articles. (DOCX)

S3 Table Proportion of reported associations* across domain of mental health and alcohol use behaviour. (DOCX)

S4 Table Proportion of reported associations limited to high-quality studies. (DOCX)

S5 Table Quality assessment criteria. (DOCX)

S6 Table The frequency of corresponding factors controlled for in the selected 36 articles. (DOCX)

S1 Fig Distribution of P-value between EXT and phenotype of alcohol use behaviours. Each dot represents the mean of P-value for all items that measure the corresponding association. P-value was either extracted or calculated using available information, and was coded as missing when not available. Dots on the right side of zero indicate size of P-values for positive associations, and dots on the left side of zero indicate size of P-value for negative associations. Two red lines represent a threshold of 0.05 respectively. “Adjust” means that INT(EXT) was adjusted simultaneously. This figure illustration applies to S2–S4 Figs.
(TIF)

S2 Fig Distribution of P-value between INT and phenotype of alcohol use behaviours. (TIF)

S3 Fig Distribution of P-value between Depression and phenotype of alcohol use behaviours. (TIF)

S4 Fig Distribution of P-value between Anxiety and phenotype of alcohol use behaviours. (TIF)

S1 Text Details on exposure and outcome of 36 included articles. (DOCX)

## References

[1] World Health Organization. Global health risks: mortality and burden of disease attributable to selected major risks. Geneva, Switzerland: World Health Organization; 2009.

[2] World Health Organization. Global status report on alcohol and health 2014. 2014.

[3] Alcohol and Public Policy Group. Alcohol: no ordinary commodity—A summary of the second edition. Addiction 2010;105:769–79. 10.1111/j.1360-0443.2010.02945.x 20331569

[4] Collishaw S, Maughan B, Natarajan L, Pickles A. Trends in adolescent emotional problems in England: a comparison of two national cohorts twenty years apart. J Child Psychol Psychiatry 2010;51:885–94. 10.1111/j.1469-7610.2010.02252.x 20497281

[5] Collishaw S, Maughan B, Goodman R, Pickles A. Time trends in adolescent mental health. J Child Psychol Psychiatry 2004;45:1350–62. 10.1111/j.1469-7610.2004.00842.x 15482496

[6] Lu W. Adolescent depression: national trends, risk factors, and healthcare disparities. Am J Health Behav 2019;43:181–94. 10.5993/AJHB.43.1.15 30522576

[7] Patalay P, Gage SH. Changes in millennial adolescent mental health and health-related behaviours over 10 years: a population cohort comparison study. Int J Epidemiol 2019 10.1093/ije/dyz006. PMC6904321 30815691

[8] Potrebny T, Wiium N, Lundegard MM-I. Temporal trends in adolescents’ self-reported psychosomatic health complaints from 1980–2016: A systematic review and meta-analysis. Plos One 2017;12:e0188374 10.1371/journal.pone.0188374 29182644 PMC5705135

[9] Bor W, Dean AJ, Najman J, Hayatbakhsh R. Are child and adolescent mental health problems increasing in the 21st century? A systematic review. Aust N Z J Psychiatry 2014;48:606–16. 10.1177/0004867414533834 24829198

[10] Sher KJ. Children of alcoholics: a critical appraisal of theory and research. Chicago: University of Chicago Press; 1991.

[11] Sher KJ, Walitzer KS, Wood PK, Brent EE. Characteristics of children of alcoholics: Putative risk factors, substance use and abuse, and psychopathology. J Abnorm Psychol 1991;100:427–48. 10.1037//0021-843x.100.4.427 1757657

[12] Iacono WG, Malone SM. Developmental endophenotypes: Indexing genetic risk for substance abuse with the P300 brain event-related potential. Child Dev Perspect 2011;5:239–47. 10.1111/j.1750-8606.2011.00205.x 22247735 PMC3254094

[13] Zucker RA, Heitzeg MM, Nigg JT. Parsing the undercontrol/disinhibition pathway to substance use disorders: A multilevel developmental problem. Child Dev Perspect 2011;5:248–55. 10.1111/j.1750-8606.2011.00172.x 22116786 PMC3221325

[14] Hussong AM, Curran PJ, Chassin L. Pathways of risk for accelerated heavy alcohol use among adolescent children of alcoholic parents. J Abnorm Child Psychol 1998;26:453–66. 10.1023/a:1022699701996 9915652

[15] Hussong AM, Jones DJ, Stein GL, Baucom DH, Boeding S. An internalizing pathway to alcohol use and disorder. Psychol Addict Behav 2011;25:390–404. 10.1037/a0024519 21823762 PMC3178003

[16] Hussong AM, Ennett ST, Cox MJ, Haroon M. A systematic review of the unique prospective association of negative affect symptoms and adolescent substance use controlling for externalizing symptoms. Psychol Addict Behav 2017;31:137–47. 10.1037/adb0000247 28134539 PMC5344716

[17] Zucker RA. Anticipating problem alcohol use developmentally from childhood into middle adulthood: what have we learned? Addiction 2008;103:100–8. 10.1111/j.1360-0443.2008.02179.x 18426543 PMC2593849

[18] Delfabbro PH, Winefield HR, Winefield AH, Hammarstrom A. Mid-adolescent predictors of adult drinking levels in early adulthood and gender differences: Longitudinal analyses based on the south Australian school leavers study. J Addict 2016;2016:1489691–1489691. 10.1155/2016/1489691 27635278 PMC5011232

[19] Essau CA, Lewinsohn PM, Olaya B, Seeley JR. Anxiety disorders in adolescents and psychosocial outcomes at age 30. J Affect Disord 2014;163:125–32. 10.1016/j.jad.2013.12.033 24456837 PMC4028371

[20] Edwards AC, Latendresse SJ, Heron J, Bin Cho S, Hickman M, Lewis G, et al Childhood internalizing symptoms are negatively associated with early adolescent alcohol use. Alcohol-Clin Exp Res 2014;38:1680–8. 10.1111/acer.12402 24848214 PMC4047162

[21] Maggs JL, Patrick ME, Feinstein L. Childhood and adolescent predictors of alcohol use and problems in adolescence and adulthood in the National Child Development Study. Addiction 2008;103:7–22. 10.1111/j.1360-0443.2008.02173.x 18426537

[22] Merline A, Jager J, Schulenberg JE. Adolescent risk factors for adult alcohol use and abuse: stability and change of predictive value across early and middle adulthood. Addiction 2008;103:84–99. 10.1111/j.1360-0443.2008.02178.x 18426542 PMC2649657

[23] Niemela S, Sourander A, Poikolainen K, Helenius H, Sillanmaki L, Parkkola K, et al Childhood predictors of drunkenness in late adolescence among males: a 10-year population-based follow-up study. Addiction 2006;101:512–21. 10.1111/j.1360-0443.2006.01381.x 16548931

[24] Hill KG, Hawkins JD, Bailey JA, Catalano RF, Abbott RD, Shapiro VB. Person–environment interaction in the prediction of alcohol abuse and alcohol dependence in adulthood. Drug Alcohol Depend 2010;110:62–9. 10.1016/j.drugalcdep.2010.02.005 20299164 PMC2885447

[25] Fanti KA, Henrich CC. Trajectories of pure and co-occurring internalizing and externalizing problems from age 2 to age 12: Findings from the national institute of child health and human development study of early child care. Dev Psychol 2010;46:1159–1175. 10.1037/a0020659 20822230

[26] Willner CJ, Gatzke-Kopp LM, Bray BC. The dynamics of internalizing and externalizing comorbidity across the early school years. Dev Psychopathol 2016;28:1033–52. 10.1017/S0954579416000687 27739391 PMC5319409

[27] Colder CR, Scalco M, Trucco EM, Read JP, Lengua LJ, Wieczorek WF, et al Prospective associations of internalizing and externalizing problems and their co-occurrence with early adolescent substance use. J Abnorm Child Psychol 2013;41:667–677. 10.1007/s10802-012-9701-0 23242624 PMC3640685

[28] Dyer ML, Easey KE, Heron J, Hickman M, Munafò MR. Associations of child and adolescent anxiety with later alcohol use and disorders: a systematic review and meta-analysis of prospective cohort studies. Addiction 2019;114:968–82. 10.1111/add.14575 30891835 PMC6563455

[29] Schulenberg JE, Maggs JL. Destiny matters: distal developmental influences on adult alcohol use and abuse. Addiction 2008;103:1–6. 10.1111/j.1360-0443.2008.02172.x. 18426536

[30] Masten AS, Faden VB, Zucker RA, Spear LP. Underage drinking: a developmental framework. Pediatrics 2008;121:S235–51. 10.1542/peds.2007-2243A 18381492

[31] Bevilacqua L, Hale D, Barker ED, Viner R. Conduct problems trajectories and psychosocial outcomes: a systematic review and meta-analysis. Eur Child Adolesc Psychiatry 2018;27:1239–60. 10.1007/s00787-017-1053-4 28983792

[32] Bucholz KK, McCutcheon VV, Agrawal A, Dick DM, Hesselbrock VM, Kramer JR, et al Comparison of parent, peer, psychiatric, and cannabis use influences across stages of offspring alcohol involvement: Evidence from the COGA prospective study. Alcohol Clin Exp Res 2017;41:359–68. 10.1111/acer.13293 28073157 PMC5272776

[33] Cheng TC, Lo CC. Social risk and protective factors in adolescents’ reduction and cessation of alcohol use. Subst Use Misuse 2017;52:916–28. 10.1080/10826084.2016.1267220 28426363

[34] Colder CR, Shyhalla K, Frndak SE. Early alcohol use with parental permission: Psychosocial characteristics and drinking in late adolescence. Addict Behav 2018;76:82–7. 10.1016/j.addbeh.2017.07.030 28772246 PMC5614833

[35] Deutsch AR, Wood PK, Slutske WS. Developmental etiologies of alcohol use and their relations to parent and peer influences over adolescence and young adulthood: A genetically informed approach. Alcohol Clin Exp Res 2017;41:2151–62. 10.1111/acer.13506 29083505 PMC5711546

[36] Lutz HR, Mcclure K, Armstrong S. Social problem solving and adolescent alcohol use within the context of well-established risk factors for adolescent alcohol use. J Child Adolesc Subst Abuse 2017;26:229–241. 10.1080/1067828X.2017.1292977.

[37] Trucco EM, Colder CR, Wieczorek WF, Lengua LJ, Hawk LW. Early adolescent alcohol use in context: How neighborhoods, parents and peers impact youth. Dev Psychopathol 2014;26:425–36. 10.1017/S0954579414000042 24621660 PMC4073105

[38] Britton A, Ben-Shlomo Y, Benzeval M, Kuh D, Bell S. Life course trajectories of alcohol consumption in the United Kingdom using longitudinal data from nine cohort studies. BMC Med 2015;13 10.1186/s12916-015-0267-x 25858476 PMC4351673

[39] Deighton J, Lereya T, Patalay P, Casey P, Humphrey N, Wolpert M. Mental health problems in young people, age 11 to 14: Results from the first HeadStart annual survey of 30,000 children. 2018.

[40] Fernandez Castelao C, Kröner-Herwig B. Different trajectories of depressive symptoms in children and adolescents: Predictors and differences in girls and boys. J Youth Adolesc 2013;42:1169–82. 10.1007/s10964-012-9858-4 23160660 PMC3714554

[41] Hicks BM, Blonigen DM, Kramer MD, Krueger RF, Patrick CJ, Iacono WG, et al Gender differences and developmental change in externalizing disorders from late adolescence to early adulthood: A longitudinal twin study. J Abnorm Psychol 2007;116:433–47. 10.1037/0021-843X.116.3.433 17696699 PMC2242627

[42] Zucker RA. Alcohol use and the alcohol use disorders: A developmental-biopsychosocial systems formulation covering the life course In: Cicchetti D, Cohen DJ, editors. Dev. Psychopathol., John Wiley & Sons, Inc.; 2015, p. 620–56. 10.1002/9780470939406.ch17.

[43] Stone AL, Becker LG, Huber AM, Catalano RF. Review of risk and protective factors of substance use and problem use in emerging adulthood. Addict Behav 2012;37:747–75. 10.1016/j.addbeh.2012.02.014 22445418

[44] Minimum legal drinking age in 190 countries 2016. https://drinkingage.procon.org/view.resource.php?resourceID=004294 (accessed June 26, 2019).

[45] Moher D. Preferred reporting items for systematic reviews and meta-analyses: The PRISMA statement. Ann Intern Med 2009;151:264 10.7326/0003-4819-151-4-200908180-00135 19622511

[46] Berg NJ, Kiviruusu OH, Lintonen TP, Huurre TM. Longitudinal prospective associations between psychological symptoms and heavy episodic drinking from adolescence to midlife. Scand J Public Health 2018 10.1177/1403494818769174. 29644935

[47] Kendler KS, Gardner CO, Edwards AC, Dick DM, Hickman M, MacLeod J, et al Childhood risk factors for heavy episodic alcohol use and alcohol problems in late adolescence: A Marginal Structural Model analysis. J Stud Alcohol Drugs 2018;79:370–9. 10.15288/jsad.2018.79.370 29885144 PMC6005251

[48] Soloski KL. Self-medication hypothesis and family socialization theory: Examining independent and common mechanisms responsible for binge drinking. Fam Process 2018;x 10.1111/famp.12403. 30357804

[49] Hoyland MA, Rowatt WC, Latendresse SJ. Prior delinquency and depression differentially predict conditional associations between discrete patterns of adolescent religiosity and adult alcohol use patterns. Subst Abuse Res Treat 2017;10 10.1177/1178221816686060. PMC5398377 28469423

[50] Squeglia L M, Ball T M, Jacobus J, Brumback T, McKenna B S, Nguyen-Louie T T, et al Neural predictors of initiating alcohol use during adolescence. Am J Psychiatry 2017;174:172–85. 10.1176/appi.ajp.2016.15121587 27539487 PMC5288131

[51] Edwards AC, Gardner CO, Hickman M, Kendler KS. A prospective longitudinal model predicting early adult alcohol problems: evidence for a robust externalizing pathway. Psychol Med 2016;46:957–68. 10.1017/S0033291715002457 26670459 PMC4801516

[52] Quinn PD, Pettersson E, Lundström S, Anckarsäter H, Långström N, Gumpert CH, et al Childhood attention-deficit/hyperactivity disorder symptoms and the development of adolescent alcohol problems: A prospective, population-based study of Swedish twins. Am J Med Genet B Neuropsychiatr Genet 2016;171:958–70. 10.1002/ajmg.b.32412 26714985 PMC5300044

[53] Savage JE, Kaprio J, Korhonen T, Pulkkinen L, Rose RJ, Verhulst B, et al The effects of social anxiety on alcohol and cigarette use across adolescence: results from a longitudinal twin study in finland. Psychol Addict Behav 2016;30:462–74. 10.1037/adb0000183 27322804 PMC4916858

[54] Swift W, Slade T, Carragher N, Coffey C, Degenhardt L, Hall W, et al Adolescent predictors of a typology of DSM-5 Alcohol Use Disorder symptoms in young adults derived by latent class analysis using data from an Australian cohort study. J Stud Alcohol Drugs 2016;77:757–65. 10.15288/jsad.2016.77.757 27588534

[55] Thompson KD, Leadbeater BJ, Ames ME. Reciprocal effects of internalizing and oppositional defiance symptoms on heavy drinking and alcohol-related harms in young adulthood. Subst Abuse Res Treat 2016;9:21–31. 10.4137/SART.S33928. PMC4723048 26819553

[56] Cook EC, Pflieger JC, Connell AM, Connell CM. Do specific transitional patterns of antisocial behavior during adolescence increase risk for problems in young adulthood? J Abnorm Child Psychol 2015;43:95–106. 10.1007/s10802-014-9880-y 24893667 PMC4256141

[57] Hyun-Jin Jun, Paul Sacco, Charlotte Lyn Bright, Camlin Elizabeth A. S. Relations among internalizing and externalizing symptoms and drinking frequency during adolescence. Subst Use Misuse 2015;50:1814–25. 10.3109/10826084.2015.1058826 26646723 PMC4757907

[58] Pesola F, Shelton KH, Heron J, Munafò M, Hickman M, Van Den Bree MBM. The developmental relationship between depressive symptoms in adolescence and harmful drinking in emerging adulthood: The role of peers and parents. J Youth Adolesc 2015;44:1752–66. 10.1007/s10964-015-0295-z 25976526 PMC4961250

[59] Virtanen P, Nummi T, Lintonen T, Westerlund H, Hägglöf B, Hammarström A. Mental health in adolescence as determinant of alcohol consumption trajectories in the Northern Swedish Cohort. Int J Public Health 2015;60:335–42. 10.1007/s00038-015-0651-5 25609507

[60] Edwards A C, Joinson C, Dick D M, Kendler K S, Macleod J, Munafo M, et al The association between depressive symptoms from early to late adolescence and later use and harmful use of alcohol. Eur Child Adolesc Psychiatry 2014;23:1219–30. 10.1007/s00787-014-0600-5 25130265 PMC4246124

[61] Tina Kretschmer, Matthew Hickman, Rita Doerner, Alan Emond, Glyn Lewis, John Macleod, et al Outcomes of childhood conduct problem trajectories in early adulthood: Findings from the ALSPAC study. Eur Child Adolesc Psychiatry 2014;23:539–49. 10.1007/s00787-013-0488-5 24197169 PMC4172989

[62] Pesola F, Shelton KH, Van Den Bree MBM. Sexual orientation and alcohol problem use among U.K. adolescents: an indirect link through depressed mood. Addict Abingdon Engl 2014;109:1072–80. 10.1111/add.12528 24612217

[63] Stanley LR, Kimberly MA, Beauvais F, Walker PS, Walker RD. Predicting an alcohol use disorder in urban American Indian youths. J Child Adolesc Subst Abuse 2014;23:101–8. 10.1080/1067828x.2012.748601 PMC8147299 34040334

[64] Meier M H, Caspi A, Houts R, Slutske W S, Harrington H, Jackson K M, et al Prospective developmental subtypes of alcohol dependence from age 18 to 32 years: Implications for nosology, etiology, and intervention. Dev Psychopathol 2013;25:785–800. 10.1017/S0954579413000175 23880392 PMC3725643

[65] Naicker K, Galambos NL, Zeng Y, Senthilselvan A, Colman I. Social, demographic, and health outcomes in the 10 years following adolescent Depression. J Adolesc Health 2013;52:533–8. 10.1016/j.jadohealth.2012.12.016 23499382

[66] Green KM, Zebrak KA, Robertson JA, Fothergill KE, Ensminger ME. Interrelationship of substance use and psychological distress over the life course among a cohort of urban African Americans. Drug Alcohol Depend 2012;123:239–48. 10.1016/j.drugalcdep.2011.11.017 22189347 PMC3319235

[67] McKenzie M, Jorm AF, Romaniuk H, Olsson CA, Patton GC. Association of adolescent symptoms of depression and anxiety with alcohol use disorders in young adulthood: findings from the Victorian Adolescent Health Cohort Study. Med J Aust 2011;195:27. 10.5694/j.1326-5377.2011.tb03262.x 21806515

[68] von Stumm S, Deary IJ, Kivimäki M, Jokela M, Clark H, Batty GD. Childhood behavior problems and health at midlife: 35-year follow-up of a Scottish birth cohort. J Child Psychol Psychiatry 2011;52:992–1001. 10.1111/j.1469-7610.2011.02373.x 21294730

[69] Bor W, McGee TR, Hayatbakhsh R, Dean A, Najman JM. Do antisocial females exhibit poor outcomes in adulthood? An Australian cohort study. Aust N Z J Psychiatry 2010;44:648–57. 10.3109/00048671003631159 20560852

[70] Huurre T, Lintonen T, Kaprio J, Pelkonen M, Marttunen M, Aro H. Adolescent risk factors for excessive alcohol use at age 32 years. A 16-year prospective follow-up study. Soc Psychiatry Psychiatr Epidemiol 2010;45:125–34. 10.1007/s00127-009-0048-y 19363578

[71] Colman I, Murray J, Abbott RA, Maughan B, Croudace TJ, Jones PB. Outcomes of conduct problems in adolescence: 40 year follow-up of national cohort. Br Med J BMJ Int Ed Lond 2009;338:208–11. 10.1136/bmj.a2981 PMC2615547 19131382

[72] Tuuli Pitkanen, Katja Kokko, Anna-Liisa Lyyra, Lea Pulkkinen. A developmental approach to alcohol drinking behaviour in adulthood: A follow-up study from age 8 to age 42. Addiction 2008;103:48–68. 10.1111/j.1360-0443.2008.02176.x 18426540

[73] Timmermans M, van Lier PAC, Koot HM. Which forms of child/adolescent externalizing behaviors account for late adolescent risky sexual behavior and substance use? J Child Psychol Psychiatry 2008;49:386–94. 10.1111/j.1469-7610.2007.01842.x 17979959

[74] Pardini D, White HR, Stouthamer-Loeber M. Early adolescent psychopathology as a predictor of alcohol use disorders by young adulthood. Drug Alcohol Depend 2007;88:S38–49. 10.1016/j.drugalcdep.2006.12.014 17257781 PMC2034413

[75] Moffitt TE, Caspi A, Harrington H, Milne BJ. Males on the life-course-persistent and adolescence-limited antisocial pathways: follow-up at age 26 years. Dev Psychopathol 2002;14:179–207. 10.1017/s0954579402001104 11893092

[76] Moffitt TE, Caspi A, Dickson N, Silva P, Stanton W. Childhood-onset versus adolescent-onset antisocial conduct problems in males: Natural history from ages 3 to 18 years. Dev Psychopathol 1996;8:399–424. 10.1017/S0954579400007161.

[77] Steele RG, Forehand R, Armistead L, Brody G. Predicting alcohol and drug use in early adulthood: the role of internalizing and externalizing behavior problems in early adolescence. Am J Orthopsychiatry 1995;65:380–8. 10.1037/h0079694 7485423

[78] Pulkkinen L, Pitkänen T. A prospective study of the precursors to problem drinking in young adulthood. J Stud Alcohol 1994;55:578–87. 10.15288/jsa.1994.55.578 7990468

[79] Pearl J. Direct and indirect effects. Proc. Seventeenth Conf. Uncertain. Artif. Intell., San Francisco: Morgan Kaufmann: 2001, p. 411–420.

[80] Del Re A. compute.es: Compute Effect Sizes. 2013.

[81] Ryan R. Heterogeneity and subgroup analyses in Cochrane Consumers and communication Group reviews: planning the analysis at protocol stage 2016.

[82] Gonnermann A, Framke T, Großhennig A, Koch A. No solution yet for combining two independent studies in the presence of heterogeneity. Stat Med 2015;34:2476–80. 10.1002/sim.6473 26040434 PMC4471592

[83] Harrison S, Jones HE, Martin RM, Lewis SJ, Higgins JPT. The albatross plot: A novel graphical tool for presenting results of diversely reported studies in a systematic review. Res Synth Methods 2017;8:281–9. 10.1002/jrsm.1239 28453179 PMC5599982

[84] CASP Cohort Study Checklist. Crit Apprais Ski Programme CASP 2018. http://www.casp-uk.net (accessed February 27, 2018).

[85] Westreich D. Berkson’s bias, selection bias, and missing data. Epidemiol Camb Mass 2012;23:159–64. 10.1097/EDE.0b013e31823b6296. PMC3237868 22081062

[86] Hammer GP, du Prel J-B, Blettner M. Avoiding bias in observational studies. Dtsch Ärztebl Int 2009;106:664–8. 10.3238/arztebl.2009.0664 19946431 PMC2780010

[87] Moffit TE, Caspi A, Dickson N, Silva P, Stanton W. Childhood-onset versus adolescent-onset antisocial conduct problems in males: Natural history from ages 3 to 18 years. Dev Psychopathol 1996;8:399–424. 10.1017/S0954579400007161.

[88] Elkins IJ, McGue M, Iacono WG. Prospective effects of attention-deficit/hyperactivity disorder, conduct disorder, and sex on adolescent substance use and abuse. Arch Gen Psychiatry 2007;64:1145–52. 10.1001/archpsyc.64.10.1145 17909126

[89] Palmer RHC, Knopik VS, Rhee SH, Hopfer CJ, Corley RC, Young SE, et al Prospective effects of adolescent indicators of behavioral disinhibition on DSM-IV alcohol, tobacco, and illicit drug dependence in young adulthood. Addict Behav 2013;38:2415–21. 10.1016/j.addbeh.2013.03.021 23685327 PMC3681900

[90] Gorka S M, Shankman S A, Olino T M, Seeley J R, Kosty D B, Lewinsohn P M. Anxiety disorders and risk for alcohol use disorders: The moderating effect of parental support. Drug Alcohol Depend 2014;140:191–7. 10.1016/j.drugalcdep.2014.04.021 24846596 PMC4076935

[91] Meisel SN, Colder CR, Bowker JC, Hussong AM. A longitudinal examination of mediational pathways linking chronic victimization and exclusion to adolescent alcohol use. Dev Psychol 2018;54:1795–1807. 10.1037/dev0000569 30058817 PMC6110953

[92] Hicks BM, Iacono WG, McGue M. Consequences of an adolescent onset and persistent course of alcohol dependence in men: Adolescent risk factors and adult outcomes. Alcohol Clin Exp Res 2010;34:819–33. 10.1111/j.1530-0277.2010.01154.x 20184563 PMC2884045

[93] Gibbons FX, Yeh H-C, Gerrard M, Cleveland MJ, Cutrona C, Simons RL, et al Early experience with racial discrimination and conduct disorder as predictors of subsequent drug use: A critical period hypothesis. Drug Alcohol Depend 2007;88:S27–37. 10.1016/j.drugalcdep.2006.12.015 17275213 PMC1868536

[94] Caspi A, Bem DJ, Elder GH. Continuities and consequences of interactional styles across the life course. J Pers 1989;57:375–406. 10.1111/j.1467-6494.1989.tb00487.x 2769561

[95] Colder CR, Frndak S, Lengua LJ, Read JP, Hawk LW, Wieczorek WF. Internalizing and externalizing problem behavior: A test of a latent variable interaction predicting a two-part growth model of adolescent substance use. J Abnorm Child Psychol 2018;46:319–30. 10.1007/s10802-017-0277-6 28229368 PMC5568518

[96] Frojd S, Ranta K, Kaltiala-Heino R, Marttunen M. Associations of social phobia and general anxiety with alcohol and drug use in a community sample of adolescents. Alcohol Alcohol 2011;46:192–9. 10.1093/alcalc/agq096 21245062

[97] Clerkin EM, Barnett N. The separate and interactive effects of drinking motives and social anxiety symptoms in predicting drinking outcomes. Addict Behav 2012;37:674–7. 10.1016/j.addbeh.2012.01.005 22365887 PMC3307890

[98] Caldwell TM, Rodgers B, Jorm AF, Christensen H, Jacomb PA, Korten AE, et al Patterns of association between alcohol consumption and symptoms of depression and anxiety in young adults. Addiction 2002;97:583–94. 10.1046/j.1360-0443.2002.00092.x 12033659

[99] O’Donnell K, Wardle J, Dantzer C, Steptoe A. Alcohol consumption and symptoms of depression in young adults from 20 countries. J Stud Alcohol 2006;67:837–40. 10.15288/jsa.2006.67.837 17061000

[100] Skogen JC, Harvey SB, Henderson M, Stordal E, Mykletun A. Anxiety and depression among abstainers and low-level alcohol consumers. The Nord-Trøndelag Health Study. Addict Abingdon Engl 2009;104:1519–29. 10.1111/j.1360-0443.2009.02659.x. 19686521

[101] Richmond RC, Al-Amin A, Smith GD, Relton CL. Approaches for drawing causal inferences from epidemiological birth cohorts: a review. Early Hum Dev 2014;90:769–80. 10.1016/j.earlhumdev.2014.08.023 25260961 PMC5154380

[102] Gage SH, Munafò MR, Smith GD. Causal inference in Developmental Origins of Health and Disease (DOHaD) research. Annu Rev Psychol 2016;67:567–85. 10.1146/annurev-psych-122414-033352 26442667

[103] Gunasekara FI, Richardson K, Carter K, Blakely T. Fixed effects analysis of repeated measures data. Int J Epidemiol 2014;43:264–269. 10.1093/ije/dyt221 24366487

[104] Fergusson DM, Boden JM, Horwood LJ. Tests of causal links between alcohol abuse or dependence and major depression. Arch Gen Psychiatry 2009;66:260–6. 10.1001/archgenpsychiatry.2008.543 19255375

[105] Pape H, Norström T. Associations between emotional distress and heavy drinking among young people: A longitudinal study. Drug Alcohol Rev 2016;35:170–6. 10.1111/dar.12290 26094994

[106] Wang J, Patten SB. Alcohol consumption and major depression: Findings from a follow-up study. Can J Psychiatry 2001;46:632–8. 10.1177/070674370104600708 11582825

[107] Dong Y, Peng C-YJ. Principled missing data methods for researchers. SpringerPlus 2013;2 10.1186/2193-1801-2-222. PMC3701793 23853744

[108] Little RJA, Rubin DB. Statistical analysis with missing data. Second Edition Chichester: Willey; 2002.

[109] White IR, Royston P, Wood AM. Multiple imputation using chained equations: Issues and guidance for practice. Stat Med 2011;30:377–99. 10.1002/sim.4067 21225900

[110] Mostafa T, Wiggins R. The impact of attrition and non-response in birth cohort studies: a need to incorporate missingness strategies. Longitud Life Course Stud 2015;6:131–46.

[111] Molina BSG, Howard AL, Swanson JM, Stehli A, Mitchell JT, Kennedy TM, et al Substance use through adolescence into early adulthood after childhood-diagnosed ADHD: findings from the MTA longitudinal study. J Child Psychol Psychiatry 2018;59:692–702. 10.1111/jcpp.12855 29315559 PMC5985671

[112] Lichtenstein P, Pettersson E, Lundstrom S, Cederlof M. Childhood aggression and adulthood adversities: general and specific associations. Behav Genet 2017;47:658–659.

