# Supplementary material for: The association between early life mental health and alcohol use behaviours in adulthood: A systematic review
Source: PLoS One. 2020 Feb 18;15(2):e0228667. doi: 10.1371/journal.pone.0228667 (PMC7028290; doi:10.1371/journal.pone.0228667)
Supplement: S1 Table — (DOCX) [file pone.0228667.s002.docx]

| S1 Table. Key words for systematic review | | |
| --- | --- | --- |
| Operator | Domain | Key words |
| #1 | Mental health | TS=(depression$ OR “depress* symptom$” OR “depress* disorder$” OR “emotional depression$” OR “mental health” OR “mental problem$” OR “mental disorder$” OR “mental illness” OR “mood disorder$” OR anxiety OR “problem* behavio$r” OR internali* OR externali* OR “conduct* problem$” OR “emotional symptom$” OR “emotional problem$” OR “emotional disorder$” OR “behavio* problem$” OR “behavio* symptom$” OR “psychological distress” OR “psychological symptom$” OR “psychological disorder$” OR “psychological health”) |
| #2 | Alcohol consumption | TS=(“alcohol drinking” OR “alcohol* drink*” OR “alcohol intake” OR “alcohol consumption” OR “alcohol behavio$r” OR “alcohol use” OR alcoholism OR “alcohol* beverage$” OR “heavy alcohol use” OR “alcohol abuse” OR “alcohol misuse” OR “alcohol problem$” OR “alcohol-related problems” OR “alcohol use disorder$” OR “alcohol dependence” OR alcohol* OR “heavy NEAR/15 drink*” OR “drink* problem$” OR “problem drink*” OR “binge drink*” OR drinker$ OR “drinking behavio$r” OR “hazardous drink*” OR “harmful drink*” OR “extreme drinking” OR “high$intensity alcohol use” OR “high$intensity drink*” OR “drink* culture”) |
| #3 | Limit Exposure Stage | TS=(“early mental health” OR child OR childhood OR child* OR adolescence OR adolescent OR adolescen* OR teen$ OR teenager$ OR youth$ OR “young people” OR “young person” OR “young adult” OR kid$ OR boy$ OR girl$ OR pupil$ OR schoolchild OR school$age OR minor$ OR “primary school” OR “secondary school” OR “elementary school” OR “high$school” OR student$ OR juvenil*) |
| #4 | Limit Study type | TS=(“life$course” OR “life$span” OR longitudinal OR cohort$ OR prospective OR life$time OR temporal OR developmental OR trajector* OR “follow$up” OR sweep$ OR wave$ OR “panel study”) |
| #5 | #1 AND #2 AND #3 AND #4 |  |
| #6 | Exclusion | TI=(pregnant OR pregnancy OR rat$ OR animal$ OR HIV OR lesbian OR gay OR mice OR cancer OR “fetal alcohol NEAR/10 dis*” OR “fetal alcohol NEAR/10 syndrom$” OR “alcoholic NEAR/10 liver disease” OR “neonatal” OR “prenatal”) |
