# Supplementary material for: The association between early life mental health and alcohol use behaviours in adulthood: A systematic review
Source: PLoS One. 2020 Feb 18;15(2):e0228667. doi: 10.1371/journal.pone.0228667 (PMC7028290; doi:10.1371/journal.pone.0228667)
Supplement: S2 Table — (DOCX) [file pone.0228667.s003.docx]

S2 Table. Extracted associations for each exposure-outcome set in 36 articles

| Study | Exposure and outcome | Association (β,SE,95%CI,*P*-value)^#^ | Sex dif^§^ | Model^&^ | Covariates | QA* |
| --- | --- | --- | --- | --- | --- | --- |
| Berg et al. (2018)[1] | INT (15.9)-H/P Drinking (22) | Female: 0.07,NR,p<0.05  Male: 0.10,NR,p<0.01 | No | Cross-lagged | Parental social economic position (16), basic education, marital and parental status, smoking, and unemployment (22, 32, 42) | 6 |
| Kendler et al. (2018)[2] | EXT (13.5)- H/P Drinking(HD) (20) | 0.024 (-0.006,0.054) | NE | MSM | Sex, parental socioeconomic status before age 8, parental alcohol problems (from pregnancy to age 12), sensation seeking at age 13.5 and 18, peer group deviance at age 12.5 and 17.5, lack of parental monitoring at age 13.5 and 15.5 | 6 |
|  | EXT (15.5)- H/P Drinking(HD) (20) | 0.042 (0.007,0.077) |  |  |  |  |
|  | EXT (13.5)- H/P Drinking(PD) (20) | 0.000 (-0.028, 0.028) |  |  |  |  |
|  | EXT (15.5)- H/P Drinking(PD) (20) | 0.078 (0.038, 0.118) |  |  |  |  |
| Soloski et al. (2018)[3] | DEP (14.9)- H/P Drinking (21.6) | -0.09 (0.04) | No | Cross-lagged | Age, sex, race, ethnicity, number of parents living in the household with the adolescent, parent alcohol use, number of three closest friends who used alcohol | 5 |
| Hoyland et al. (2017)[4] | DEP (15.6)- H/P Drinking (29.6) | Problem vs low-intake:  OR 1.139, p=0.361  Non-problem vs low-intake:  OR 0.532, p=0.129  Abstainer vs low-intake:  OR 0.929, p=0.482  Problem vs abstainers:  OR 1.059, P=0.681  Nonproblem vs abstainers:  OR 0.782, p=0.019  Problem vs nonproblem:  OR 1.353, p=0.023 | NE | Logistic reg | Age, race, and sex, wave one alcohol use and Catholic/Protestant identification, delinquency, delinquency*depression | 3 |
| Squeglia et al. (2017)[5] | EXT (13)-AC (18) | NR, positive, p=0.007 | NE | Random forest analyses | A series of demographic, neuropsychological and neuroimaging variables, but not sex | 4 |
| Edwards et al. (2016)[6] | EXT (11.8)- H/P Drinking (20) | 0.031, NR, p<0.05 | No | SEM | Sex, parental SES, paternal AP, maternal AP, good peer relationships 11.8, low parental monitoring12.8, peer group deviance12.8, extraversion13.6, low conscientiousness13.6, sensation seeking13.6, conduct disorder15.6, major depression symptoms16.6, peer group deviance17.6, stressful life events17.6, illicit substance use17.6 (alcohol problem17.6, sensation seeking18, illicit substance use18) | 5 |
|  | EXT (15.6)- H/P Drinking (20) | 0.146, NR, p<0.05 |  |  |  |  |
|  | DEP (16.6)- H/P Drinking (20) | 0.035, NR, p<0.05 |  |  |  |  |
| Quinn et al.  (2016)[7] | EXT (15)- H/P Drinking (18) | 0.25 (0.19,0.32) | NE | OLS | Sex | 3 |
| Savage et al. (2016)[8] | ANX (12)-AC(22) | -0.1 (0.04), p<0.05 | No | Pearson Correlation | None | 4 |
| Swift et al. (2016)[9] | Onset of EXT (14.5~17)-AUD(24) | OR(95%)  Moderate vs mild: 0.96 (0.60,1.6)  Severe vs mild: 3.2 (1.5,6.8)  Severe vs moderate: 3.3 (1.6,7.1) | NE | Multi-nomial logistic reg | Geographical location, sex, frequency of parental drinking, frequency of parental smoking, parental education, and parental divorce/separation, adolescent indices: age at alcohol use onset<15 years, frequent/binge alcohol use, alcohol use problems,daily cigarette smoking, weekly cannabis use, persisting symptoms of anxiety/depression, antisocial behaviors | 6 |
|  | Onset of INT (14.5~17)-AUD(24) | Moderate vs mild: 1.9 (1.2,3.1)  Severe vs mild: 2.5 (1.3,5.0)  Severe vs moderate: 1.3 (0.6,3.2) |  |  |  |  |
| Cook et al. (2015)[10] | EXT across time (13.9~14.9)- H/P Drinking (20.3) | Reference group: non-ASB, OR  Agg Stable (female): 1.09(0.73,1.63)  Theft Stable(female): 1.79(1.36,2.42)  Serious Stable(female): 1.23(0.65,2.28)  Escalators(female): 2.19(1.48,3.23)  De-Escalators(female): 1.30(0.97,1.74)  Agg Stable*male: 1.93(1.19,3.12)  Theft Stable*male: 1.32(0.85,2.01)  Serious Stable*male: 2.52(1.20,5.28)  Escalators*male: 0.89(0.53,1.52)  De-Escalators*male: 1.19(0.83,1.72) | Yes | Logistic reg | Sex, adolescents’ age as indicated by grade, race/ethnicity (dummy coded with ‘White’ as the reference group), and poverty status | 5 |
| Jun et al.  (2015)[11] | EXT(15)-AC(18) | No association for male, coefficient NR  No association for female, coefficient NR | No | Cross-lagged | Race/ethnicity of subjects, and salary and educational level of primary caregivers | 3 |
|  | INT(15)-AC(18) | No association for male, coefficient NR  No association for female, coefficient NR |  |  |  |  |
| Pesola et al. (2015)[12] | DEP(14)- H/P Drinking (19) | 0.049 (0,0.098) | NE | SEM | Sex, financial difficulties, family education level, parents’ alcohol consumption, and parents’ depression, SDQ conducting problem, earlier deviant peers at 13, earlier alcohol use at 13 | 5 |
| Thompson et al. (2016)[13] | EXT(16/17)-  H/P Drinking(HD)(18/19) | 0.08,p<0.001 | No | Cross-lagged | Sex, mother’s education | 3 |
|  | EXT(16/17)-  H/P Drinking (PD)(18/19) | 0.10, p<0.001 |  |  |  |  |
|  | INT(16/17)-  H/P Drinking (HD)(18/19) | No association, NR |  |  |  |  |
|  | INT(16/17)-  H/P Drinking (PD)(18/19) | 0.10,p<0.001 |  |  |  |  |
| Virtanen et al.(2015)[14] | DEP(16)-AC(16~45) | Reference group: compliant group, OR(95%)  Late onset low: 1.07(0.45,2.50)  Ordinary:1.41(0.74,2.72)  Early onset low:2.37(1.14,4.93)  Early onset moderate:2.46(1.31,4.64)  Early onset high:2.86(1.45,5.66) | No | Multi-nomial logistic reg | Sex, parental social class | 4 |
|  | ANX(16)-AC(16~45) | Reference group: compliant group, OR(95%)  Late onset low: 1.54(0.72,3.32)  Ordinary:1.97(1.08,3.60)  Early onset low:2.43(1.21,4.88)  Early onset moderate:2.84(1.56,5.15)  Early onset high:3.59(1.89,6.82) |  |  |  |  |
| Edwards et al. (2014)[15] | DEP(12~17)- H/P Drinking (18.5) | Intercept  0.05(-0.07,0.16), p=0.426 for male  0.04(-0.05,0.13), p=0.610 for female  Slope  -0.03(-0.17,0.10),p=0.432 for male  0.15(0.04,0.25),p=0.007 for female | Yes | Growth Curve Model+OLS | Maternal education, parity and tenure, smoking 12, alcohol 12, cannabis 9, EPDS 11, conduct problems11, bullying 13, smoking, cannabis and alcohol 13 | 5 |
| Kretschmer et al. (2014)[16] | EXT(4~13)- H/P Drinking (18) | Reference group: low, OR(95%)  Childhood-limited(CL): 0.86(0.39,1.86)  Adolescence-onset(AO): 1.68(0.89,3.20)  Early onset persistent(EOP):1.91(1.21,3.01)  Reference group:CL  EOP:2.22(0.95,5.26)  AO:1.96(0.70,5.50)  EOP vs AO: 1.14(0.55,2.33) | NE | Logistic reg | Socio-economic status, marital status/cohabitation, maternal education, and age of the mother when first pregnant, drinking during pregnancy and maternal family history of alcohol use, smoking during pregnancy, any maternal contact with the police during child’s first 4 years of life, childbirth weight, gestational age, parity and a single indicator for any birth complications, language development, child temperament at 24 months postpartum, maternal depression, anxiety at 32 weeks antenatal and 8 weeks postnatal, harsh parenting at 24 months and partner emotional and/or physical cruelty to the mother during child’s first 4 years of life. low emotional and practical support for the mother during child’s first 4 years of life. Indication of child head injury during child’s first 4 years of life. Maternal attitude toward the child at 33 months postpartum. | 4 |
| Pesola et al.  (2014)[17] | DEP(16)- H/P Drinking (18) | 0.06, p=0.001 | No | SEM | Family environment (i.e. parental drinking and depression, collected at 12 years) and socio-economic status, earlier depressed mood and earlier alcohol problem use at age 16 | 5 |
| Stanley et al. (2014)[18] | EXT(11.7)-AUD(19.7) | OR(95%) 1.05(0.99,1.12) | NE | Logistic reg | Sex, income, alcoholic mother, alcoholic father, family cohesion, family conflict, likes school, early alcohol initiation, and Internalising behaviours accordingly | 5 |
|  | INT(11.7)-AUD(19.7) | OR(95%) 0.96(0.91,1.02) |  |  |  |  |
| Meier et al.  (2013)[19] | EXT(5-11)-AUD onset(18~32) | 1.04(0.91,1.20),p=0.53 | NE | Logistic reg | Sex | 4 |
|  | INT(5-11)-AUD onset(18~32) | 0.95(0.82,1.09),p=0.43 |  |  |  |  |
|  | EXT(11-18)-AUD onset(18-32) | 3.08(2.24,4.24),p<0.001 |  |  |  |  |
|  | DEP(11-18)-AUD onset(18-32) | 2.31(1.65,3.24),p<0.001 |  |  |  |  |
|  | ANX(11-18)-AUD onset(18-32) | 1.57(1.17,2.10),p=0.002 |  |  |  |  |
|  | EXT(5-11)-AUD traj(18~32) | Persistent vs develop limited:  1.10(0.76,1.58),p=0.62 |  |  |  |  |
|  | INT(5-11)-AUD traj (18~32) | 1.21(0.82,1.81),p=0.34 |  |  |  |  |
|  | EXT(11-18)-AUD traj (18-32) | 2.11(0.96,4.64),p=0.06 |  |  |  |  |
|  | DEP(11-18)-AUD traj (18-32) | 3.49(1.48,8,25),p=0.004 |  |  |  |  |
|  | ANX(11-18)-AUD traj (18-32) | 2.30(1.02,5.22),p=0.04 |  |  |  |  |
|  | EXT(5-11)-AUD traj(18~32) | Adult onset vs never diagnosed  1.08(0.81,1.45),p=0.59 |  |  |  |  |
|  | INT(5-11)-AUD traj (18~32) | 1.08(0.80,1.46),p=0.62 |  |  |  |  |
|  | EXT(11-18)-AUD traj (18-32) | 1.50(0.71,3.19),p=0.29 |  |  |  |  |
|  | DEP(11-18)-AUD traj (18-32) | 1.90(0.92,3.96),p=0.09 |  |  |  |  |
|  | ANX(11-18)-AUD traj (18-32) | 1.55(0.82,2.93),p=0.18 |  |  |  |  |
| Naicker et al. (2013)[20] | DEP(16/17)-H/P Drinking (18/19) | 2.70(1.20,6.07) | NE | Logistic reg | Sex and adolescent socioeconomic status | 4 |
|  | DEP(16/17)-H/P Drinking (20/21) | 1.47(0.67,3.25) |  |  |  |  |
|  | DEP(16/17)-H/P Drinking (22/23) | 1.39(0.46,4.21) |  |  |  |  |
|  | DEP(16/17)-H/P Drinking (24/25) | 2.14(0.72,6.44) |  |  |  |  |
|  | DEP(16/17)-H/P Drinking (26/27) | 1(0.33,3.1) |  |  |  |  |
|  | DEP(16/17)-H/P Drinking (18-27) | 1.78(1.10,2.87) |  | Generalized Linear Mixed Model |  |  |
| Green et al.  (2012)[21] | INT(16)-AC(32/33) | -0.311(0.147),p<0.05 for male  -0.023(0.111),p>0.05 for female | Yes | SEM | Mother's psychological distress at childhood and adolescence; low social economic status, mother's rating of psychological distress, teacher's rating of psychological distress, poor school performance, poor classroom behaviour, mother's substance use at adolescence, later psychological distress and marijuana/cocaine use | 5 |
| McKenzie et al.  (2011)[24] | INT(15.5-17.4)-AUD(24) | Reference group: 0 waves OR(95%)  1-2 waves: 1.3(1.2-1.4),p<0.001  >2 waves: 1.9(1.7-2.0),p<0.001 | NE | Logistic reg | Adolescent alcohol use, tobacco use, sex, school location, country of birth, parental education, marital status, parental tobacco and alcohol use | 8 |
| Stumm et al. (2011)[25] | EXT(9.7)-AC(46~52) | Drinking cessation(binary):  1.10(0.91,1.33) for male  1.08(0.84,1.40) for female  Four or more drinks(continuous):  -0.01(-0.09,0.05) for male  0.01(-0.08,0.10) for female  Hangovers(continuous):  -0.00(-0.05,0.04) for male  0.02(-0.03,0.08) for female  Drinking frequency(categorical):  Reference group: drinking on special occasions  MALE  Every day: 0.96(0.78,1.19)  Most days: 0.85(0.70,1.03)  Weekends: 0.94(0.80,1.11)  <once a week: 0.93(0.77,1.12)  FEMALE  Every day: 1.27(0.94,1.72)  Most days: 0.98(0.75,1.28)  Weekends: 1.01(0.84,1.21)  <once a week: 1.11(0.90,1.36)  Alcohol amount(categorical):  Reference group: light drinkers  MALE  Moderate drinker: 1.01(0.88,1.16)  Extreme drinker: 0.99(0.88,1.11)  FEMALE  Moderate drinker: 0.95(0.78,1.16)  Extreme drinker: 1.27(1.06,1.52) | NE | OLS(continuous); Logistic reg(binary); Multinomial logistic reg(categorical) | Age, intelligence, social class of origin, and educational qualification | 3 |
|  | INT(9.7)-AC(46~52) | Drinking cessation(binary):  1.09(0.90,1.33) for male  1.00(0.84,1.19) for female  Four or more drinks(continuous):  -0.08(-0.18,-0.06) for male  -0.06(-0.14,-0.02) for female  Hangovers(continuous):  -0.05(-0.10,-0.01) for male  -0.01(-0.04,0.02) for female  Reference group: drinking on special occasions  MALE  Every day: 0.99(0.82,1.21)  Most days: 0.88(0.75,1.04)  Weekends: 0.92(0.79,1.07)  <once a week: 0.94(0.78,1.12)  FEMALE  Every day: 0.88(0.69,1.13)  Most days: 1.00(0.88,1.15)  Weekends: 0.92(0.83,1.03)  <once a week: 1.05(0.92,1.18)  Alcohol amount(categorical):  Reference group: light drinkers  MALE  Moderate drinker: 0.80(0.69,0.92)  Extreme drinker: 0.94(0.85,1.05)  FEMALE  Moderate drinker: 0.91(0.81,1.02)  Extreme drinker: 0.90(0.79,1.02) |  |  |  |  |
| Bor et al. (2010)[26] | EXT(5-14)-AC(21) | Reference group:  Unclassified group for EXT  Non-drinker for AC  Male-1-6 drinks per occasion  CL: 0.9(0.5,1.7)  AL:1.0(0.5,2.2)  LCP:1.0(0.2,4.5)  Female-1-6 drinks per occasion  CL: 1.9(0.9,4.1)  AL:0.9(0.5,1.7)  LCP:0.4(0.2,0.9)  Male->6 drinks per occasion  CL: 1.1(0.6,2.0)  AL:1.7(0.8,3.6)  LCP:2.6(0.6,11.2)  Female->6 drinks per occasion  CL: 1.6(0.6,3.8)  AL:1.3(0.6,2.7)  LCP:0.4(0.1,1.2) | NE | Multinomial logistic reg | None | 2 |
| Hill et al.  (2010)[27] | EXT(14/15)-AUD(27) | Alcohol abuse:0.086,p=0.060  Alcohol dependence: 0.076,p=0.092 | NE | OLS | Ethnicity, sex and poverty, past-month drinking at age 12, Family management—ages 11–14, Behavioral inhibition/anxiety (BI/A)—ages 14–15, Behavioral disinhibition/anxiety (BDI)—ages 14–15 accordingly | 5 |
|  | ANX(14/15)-AUD(27) | Alcohol abuse: -0.015,p=0.723  Alcohol dependence: 0.008,p=0.837 |  |  |  |  |
| Huurre et al. (2010)[28] | DEP(16)-H/P Drinking (32) | Reference: Low depression  Male  Middle: 1.69(1.01,2.84),p=0.048  High: 1.77(1.01,3.11),p=0.045  Female  Middle:1.34(0.73,2.46),p=0.34  High:0.96(0.49,1.86),p=0.892 | NE | Logistic reg | Parental social class, school performance, parental divorce, relationship with mother, relationship with father, parental trust, self-esteem, impulsiveness, spent leisure-time daily among friends, dating experience, drinking habit, smoking habit, problems with the law. | 4 |
| Colman et al. (2009)[29] | EXT(13-15)-H/P Drinking (43-53) | Reference: no EXT group  Mild: 1.4(1.0,1.9)  Severe:1.2(0.7,2.1) | No | Ordinal logistic reg | Sex, father’s social class, cognitive ability, and depression-anxiety in adolescence | 3 |
| Maggs et al. (2008)[30] | EXT(7)-AC(23) | 2.17(0.84) for man, p<0.05  0.27(0.30) for woman, p>0.05 | NE | OLS/Logistic reg | Social class and parents’ educational level, parents reading with the child (at age 7), academic ability, academic test scores, Social maladjustment, Externalising behaviour (EB) at ages 7 and 11 accordingly, Internalising behaviour (IB) at ages 7 and 11 accordingly | 5 |
|  | INT(7)-AC(23) | -3.66(0.76) for man, p<0.001  -0.59(0.26) for woman, p<0.05 |  |  |  |  |
|  | EXT(11)-AC(23) | 2.72(0.91) for man, p<0.01  0.09(0.33) for woman, p>0.05 |  |  |  |  |
|  | INT(11)-AC(23) | -2.54(0.82) for man, p<0.01  -0.01(0.28) for woman, p>0.05 |  |  |  |  |
|  | EXT(7)-AC(33) | 2.41(0.75) for man, p<0.01  0.70(0.32) for woman, p<0.05 |  |  |  |  |
|  | INT(7)-AC(33) | -3.08(0.67) for man, p<0.001  -0.83(0.28) for woman, p<0.01 |  |  |  |  |
|  | EXT(11)-AC(33) | 1.49 (0.80) for man, p>0.05  -0.10(0.35) for woman, p>0.05 |  |  |  |  |
|  | INT(11)-AC(33) | -2.77(0.72) for man, p<0.001  -0.76(0.30) for woman, p<0.05 |  |  |  |  |
|  | EXT(7)-H/P Drinking (42) | 1.10, NR for man, p>0.05  1.54 (1.14,2.09) for woman, p<0.01 |  |  |  |  |
|  | INT(7)-H/P Drinking (42) | 0.91, NR for man, p>0.05  0.89, NR for woman, p>0.05 |  |  |  |  |
|  | EXT(11)-H/P Drinking (42) | 1.30, NR for man, p>0.05  1.59 (1.07,2.36) for woman, p<0.05 |  |  |  |  |
|  | INT(11)-H/P Drinking (42) | 0.78, NR for man, , p>0.05  1.04, NR for woman, p>0.05 |  |  |  |  |
| Pitkanen et al. (2008)[31] | ANT(8)-H/P Drinking (20) | 0.06 for male, p>0.05  0,04 for female, p>0.05 | No | OLS | Age8 social behaviour accordingly: social activity, constructiveness, compliance, aggressiveness, low self-control, anxiety, school success; Age14 social behaviour accordingly: social behaviour, constructiveness, compliance, aggressiveness, low self-control, anxiety, school success | 5 |
|  | ANT(8)-H/P Drinking(PD)(27) | -0.15 for male,p>0.05  0.09 for female,p>0.05 |  |  |  |  |
|  | ANT(8)-H/P Drinking(PD)(42) | 0.06 for male,p>0.05  -0.02 for female,p>0.05 |  |  |  |  |
|  | ANT(14)-H/P Drinking(20) | -0.24 for male, p<0.01  -0.07 for female, p>0.05 |  |  |  |  |
|  | ANT(14)-H/P Drinking(PD)(27) | 0.01 for male,p>0.05  0.00 for female,p>0.05 |  |  |  |  |
|  | ANT(14)-H/P Drinking(PD) (42) | -0.14 for male,p>0.05  -0.11 for female,p>0.05 |  |  |  |  |
|  | ANT(8)-AC(27) | 0.06 for male,p>0.05  -0.06 for female,p>0.05 |  |  |  |  |
|  | ANT(8)-AC(42) | -0.03 for male,p>0.05  -0.08 for female,p>0.05 |  |  |  |  |
|  | ANT(14)-AC(27) | -0.15 for male,p>0.05  -0.20 for female, p<0.05 |  |  |  |  |
|  | ANT(14)-AC(42) | -0.01 for male,p>0.05  -0.19 for female, p<0.05 |  |  |  |  |
|  | ANT(8)-H/P Drinking(HD)(27) | 0.15 for male,p>0.05  -0.07 for female,p>0.05 |  |  |  |  |
|  | ANT(8)-H/P Drinking(HD)(42) | -0.03 for male,p>0.05  -0.06 for female,p>0.05 |  |  |  |  |
|  | ANT(14)-H/P Drinking(HD)(27) | -0.16 for male,p>0.05  -0.13 for female, p<0.05 |  |  |  |  |
|  | ANT(14)-H/P Drinking(HD)(42) | -0.16 for male,p>0.05  -0.13 for female, p<0.05 |  |  |  |  |
|  | ANT(8)-H/P Drinking(CAGE)(27) | 0.07 for male,p>0.05  0.09 for female, p>0.05 |  |  |  |  |
|  | ANT(8)-H/P Drinking(CAGE)(42) | 0.08 for male,p>0.05  0.00 for female, p>0.05 |  |  |  |  |
|  | ANT(14)-H/P Drinking(CAGE)(27) | -0.09 for male,p>0.05  -0.03 for female,p>0.05 |  |  |  |  |
|  | ANT(14)-H/P Drinking(CAGE)(42) | -0.03 for male,p>0.05  -0.12 for female, p>0.05 |  |  |  |  |
| Timmermans et al. (2008)[32] | EXT(4-18)-H/P Drinking(CAGE)(18) | Intercept: 0.11(0.04),p<0.01  Slope: 0.95(0.42),p<0.05 | No | OLS | None | 2 |
| Pardini et al. (2007)[33] | EXT(13.9)-AUD(20.4-25.4) | Zero inflation:  1.065(0.808,1.402), p=0.656  Symptom count:  1.190(1.054,1.342), p=0.005 | NA | Poisson reg | Age, minority status, family socioeconomic status, parent alcohol/drug problems, child's prior alcohol use, child's prior alcohol problems, CD symptoms, ADHA symptoms, depression, Anxiety/withdrawal | 7 |
|  | DEP(13.9)-AUD(20.4-25.4) | Zero inflation:  0.956(0.736,1.241), p=0.736  Symptom count:  1.048(0.937,1.172), p=0.408 |  |  |  |  |
|  | ANX(13.9)-AUD(20.4-25.4) | Zero inflation:  1.079(0.806,1.445), p=0.610  Symptom count:  0.858(0.774,0.952), p=0.004 |  |  |  |  |
| Niemela et al. (2006)[34] | EXT(8)-H/P Drinking(18) | Reference: no drunkenness  Drunkenness less than weekly:  1.0(0.94,1.16)  Drunkenness once a week or more often:  1.1(1.0,1.31) | NA | Logistic reg | Non-intact family structure, hyperactive, conduct and emotional problems according to teacher’s report | 4 |
|  | INT(8)-H/P Drinking(18) | Drunkenness less than weekly:  0.8(0.71,0.90)  Drunkenness once a week or more often:  0.8(0.68,0.96) |  |  |  |  |
| Moffit et al. (2002)[35] | EXT(5-18)-AUD(26) | Reference: Unclassified group  Abstainer:  no, p>0.01  Recovery:  no, p>0.01  Life-course Persistent path:  positive, p<0.01  Adolescence-limited path:  positive, p<0.01  LCP vs AL: positive, p=0.002 | NA | ANOVA | None | 3 |
| Moffitt et al. (1996)[36] | EXT(5-18)-AUD(18) | Reference: Unclassified group  Abstainer:  no, p>0.01  Recovery:  no, p>0.01  Life-course Persistent path:  positive, p<0.01  Adolescence-limited path:  positive, p<0.01  LCP vs AL: no, p>0.05 | NA | ANOVA | None | 3 |
| Steele et al.  (1995)[37] | EXT(13.5)-AUD(19.75) | 0.087,p=0.05 | No | OLS | Sex, anxiety/conduct problem accordingly, anxiety*sex, conduct problem*sex, anxiety*conduct problem, anxiety*conduct problem*sex | 2 |
|  | ANX(13.5)-AUD(19.75) | -0.042,p>0.05 |  |  |  |  |
| Pulkkinen et al.(1994) [38] | EXT(14)-H/P Drinking(26-27) | 0.20 for male, p<0.05  No for female,p>0.05 | Yes | Path analysis | Anxiety at age 8, aggression at age 8, prosociality at age 8, school success at age 8, school success at age 14 | 3 |
|  | ANX(14)-H/P Drinking(26-27) | -0,21 for male, p<0.01  No for female,p>0.05 |  |  |  |  |
| ^#^Report in order whatever is available in the study  ^§^Sex differences of the association; NE: not explored; NA: not appliable  ^&^EXT: externalising problems; INT: internalising problems; DEP: depression/depressive symptoms; ANX; anxiety; AC: alcohol consumption; H/P drinking: heavy/problematic drinking; HD: heavy episode drinking/binge drinking; PD: problematic drinking; AUD: alcohol use disorder; OLS: ordinary least-square regression; reg: regression; ANOVA: analysis of variance; SEM: structural equation model; MSM: marginal structural model  *Quality assessment  !The acronym applied throughout the supplements. | | | | | | |
