# Supplementary material for: The association between early life mental health and alcohol use behaviours in adulthood: A systematic review
Source: PLoS One. 2020 Feb 18;15(2):e0228667. doi: 10.1371/journal.pone.0228667 (PMC7028290; doi:10.1371/journal.pone.0228667)
Supplement: S3 Table — (DOCX) [file pone.0228667.s004.docx]

S3 Table. Proportion of reported associations* across domain of mental health and alcohol use behaviour

|  | Alcohol consumption | | | Heavy/problematic drinking | | | Alcohol Use Disorder | | |
| --- | --- | --- | --- | --- | --- | --- | --- | --- | --- |
|  | positive | negative | no | positive | negative | no | positive | negative | no |
| Externalizing domain | 3/5  60% | 1/5  20% | 1/5  20% | 10/11  90.9% | 0/11  0% | 1/11  9.1% | 3/5  60% | 0/5  0% | 2/5  40% |
| Internalizing domain |  |  |  |  |  |  |  |  |  |
| Internalizing | 0/4  0% | 3/4  75% | 1/4  25% | 2/5  40% | 2/5  40% | 1/5  20% | 1/3  33.3% | 0/3  0% | 2/3  66.7% |
| Depression^#^ | 1/1  100% | 0/1  0% | 0/1  0% | 3/4  75% | 1/4  25% | 0/4  12.5% | 2/3  66.7% | 0/3  0% | 1/3  33.3% |
| Anxiety | 1/3  33.3% | 2/3  66.7% | 0/3  0% | 0/2  0% | 1/2  50% | 1/2  50% | 1/3  33.3% | 1/3  33.3% | 1/3  33.3% |

*for each exposure-outcome set, no matter how many items there were, the pair of association was counted as significant as long as one item is significant.
