# Supplementary material for: The association between early life mental health and alcohol use behaviours in adulthood: A systematic review
Source: PLoS One. 2020 Feb 18;15(2):e0228667. doi: 10.1371/journal.pone.0228667 (PMC7028290; doi:10.1371/journal.pone.0228667)
Supplement: S4 Table — (DOCX) [file pone.0228667.s005.docx]

S4 Table. Proportion of reported associations limited to high-quality studies

|  | Alcohol consumption | | | Heavy/problematic drinking | | | Alcohol Use Disorder | | |
| --- | --- | --- | --- | --- | --- | --- | --- | --- | --- |
|  | positive | negative | no | positive | negative | no | positive | negative | no |
| Externalizing domain | 4/8  50% | 0/8  0% | 4/8  50% | 12/20  60% | 0/20  0% | 8/20  40% | 3/8  37.5% | 0/8  0% | 5/8  62.5% |
| Internalizing domain |  |  |  |  |  |  |  |  |  |
| Internalizing | 0/10  0% | 8/10  80% | 2/10  20% | 2/6  33.3% | 0/6  0% | 4/6  66.7% | 4/6  66.7% | 0/6  0% | 2/6  33.3% |
| Depression | 0/0  0% | 0/0  0% | 0/0  0% | 4/8  50.0% | 1/8  12.5% | 3/8  37.5% | 0/2  0% | 0/2  0% | 2/2  100% |
| Anxiety | 0/0  0% | 0/0  0% | 0/0  0% | 0/0  0% | 0/0  0% | 0/0  0% | 0/4  0% | 1/4  25% | 0/4  0% |
