# Supplementary material for: The association between early life mental health and alcohol use behaviours in adulthood: A systematic review
Source: PLoS One. 2020 Feb 18;15(2):e0228667. doi: 10.1371/journal.pone.0228667 (PMC7028290; doi:10.1371/journal.pone.0228667)
Supplement: S5 Table — (DOCX) [file pone.0228667.s006.docx]

S9 Table. Quality assessment criteria

|  |
| --- |
| **Sample** |
| How is the representativeness of the sample?  1=not representative  2=representative of the local/whole population |
| Is the sample size large enough?  1=No (below 1000)  2=Yes (over 1000) |
| **Assessment** |
| How is the exposure measured?  1= self-report/reported by others  2= clinical interview/record linkage |
| How is the outcome measured?  1= self-report/reported by others  2= clinical interview/record linkage |
| **Confounders** |
| Has the author identified and control all important confounding factors?  1=No  2=Yes (Core confounding factors include: gender, family SES) |
| Has the author controlled for other relevant confounders or other more advanced analysis?  1=No  2=Yes |
| **Missing data** |
| Was the follow up of subjects complete enough?  1=No (attrition rate >=20% or attrition analysis shows significant biases)  2=Yes (attrition rate<20% or attrition analysis shows no significant biases) |
| Has the author taken account of the missing data?  1=No (complete case analysis or traditional missing data method)  2=Yes (advanced methods including Inverse Probability Weight, Multiple imputation, Maximum likelihood estimation) |
