# Supplementary material for: The association between early life mental health and alcohol use behaviours in adulthood: A systematic review
Source: PLoS One. 2020 Feb 18;15(2):e0228667. doi: 10.1371/journal.pone.0228667 (PMC7028290; doi:10.1371/journal.pone.0228667)
Supplement: S6 Table — (DOCX) [file pone.0228667.s007.docx]

| S10 Table. The frequency of corresponding factors controlled for in the selected 36 articles | |
| --- | --- |
| Family-related factors | Individual-related factors |
| Family socioeconomic status (19) | Demographics (26) |
| Prenatal and post-natal indicators (2) | Intrinsic trait (Personality/IQ) (8) |
| Parental education (11) | Prior mental health status (15) |
| Parental marital status (7) | Prior substance use (13) |
| Parental mental health status(8) | History of abuse/neglect (0) |
| Parental substance use (12) | School performance (6) |
| Family function (3) | Transitional life events (3) |
| Social factors | Personal belief/moral order (1) |
| Peer relationship (4) | Physical health status (1) |
| Peer substance use (4) | Self-regulation ability (1) |
|  | Victimization/bullying (2) |
|  | Risky behaviour (4) |
| *demographics includes gender, race/ethnicity, religion and own financial status; family function refers to family relations, communication, management and family support et al; transitional life events include college attendance, involvement in a relationship/marriage, pregnancy or other major life events. | |
