# Supplementary material for: The association between early life mental health and alcohol use behaviours in adulthood: A systematic review
Source: PLoS One. 2020 Feb 18;15(2):e0228667. doi: 10.1371/journal.pone.0228667 (PMC7028290; doi:10.1371/journal.pone.0228667)
Supplement: S1 Text — (DOCX) [file pone.0228667.s012.docx]

S11 Text. Details on exposure and outcome of 36 included articles

Exposure included internalizing problems (n=5), depression (n=13), anxiety (n=8) and other emotional and psychological statuses (n=4), as well as externalizing problems (n=7), conduct problems (n=10) and anti-social behaviour (n=6). Outcomes included drinking frequency (how often the participants drank alcohol, n=5), drinking quantity (how much the participants drank, usually per occasion, n=2), total drinking volume (calculated by drinking frequency x quantity, n=3), drunkenness (frequency of drunkenness or number of drunkenness occasions during a certain time period, n=8), problem drinking (featured by being involved in accidents/fights/conflicts because of drinking, n=5), harmful drinking (assessed by the CAGE/AUDIT/Michigan Alcohol Screening Test which indicate the possibility of AUD, n=13), and AUD (as defined in Diagnostic and Statistical Manual III/IV which refers to either alcohol abuse (AA) or alcohol dependence (AD), n=10).
